# Mus musculus papillomavirus 1 E8^E2 represses expression of late protein E4 in basal-like keratinocytes via NCoR/SMRT-HDAC3 co-repressor complexes to enable wart formation *in vivo*

**DOI:** 10.1128/mbio.00696-23

**Published:** 2023-06-29

**Authors:** Franziska Kuehner, Margaret Wong, Elke Straub, John Doorbar, Thomas Iftner, Richard B. S. Roden, Frank Stubenrauch

**Affiliations:** 1 Institute for Medical Virology and Epidemiology of Viral Diseases, University Hospital Tuebingen, Eberhard-Karls University Tuebingen, Tuebingen, Germany; 2 Department of Pathology, The Johns Hopkins University, Baltimore, Maryland, USA; 3 Department of Pathology, University of Cambridge, Cambridge, United Kingdom; Princeton University, Princeton, New Jersey, USA

**Keywords:** papillomavirus, E8^E2, E4, wart, productive replication

## Abstract

High-risk human papillomaviruses (PV) account for approximately 600,000 new cancers per year. The early protein E8^E2 is a conserved repressor of PV replication, whereas E4 is a late protein that arrests cells in G2 and collapses keratin filaments to facilitate virion release. While inactivation of the Mus musculus PV1 (MmuPV1) *E8* start codon (E8-) increases viral gene expression, surprisingly, it prevents wart formation in FoxN1^nu/nu^ mice. To understand this surprising phenotype, the impact of additional E8^E2 mutations was characterized in tissue culture and mice. MmuPV1 and HPV E8^E2 similarly interact with cellular NCoR/SMRT-HDAC3 co-repressor complexes. Disruption of the splice donor sequence used to generate the *E8^E2* transcript or E8^E2 mutants (mt) with impaired binding to NCoR/SMRT-HDAC3 activates MmuPV1 transcription in murine keratinocytes. These MmuPV1 E8^E2 mt genomes also fail to induce warts in mice. The phenotype of E8^E2 mt genomes in undifferentiated cells resembles productive PV replication in differentiated keratinocytes. Consistent with this, E8^E2 mt genomes induced aberrant E4 expression in undifferentiated keratinocytes. In line with observations for HPV, MmuPV1 E4-positive cells displayed a shift to the G2 phase of the cell cycle. In summary, we propose that in order to enable both expansion of infected cells and wart formation *in vivo*, MmuPV1 E8^E2 inhibits E4 protein expression in the basal keratinocytes that would otherwise undergo E4-mediated cell cycle arrest.

**IMPORTANCE:**

Human papillomaviruses (PVs) initiate productive replication, which is characterized by genome amplification and expression of E4 protein strictly within suprabasal, differentiated keratinocytes. Mus musculus PV1 mutants that disrupt splicing of the E8^E2 transcript or abolish the interaction of E8^E2 with cellular NCoR/SMRT-HDAC3 co-repressor complexes display increased gene expression in tissue culture but are unable to form warts *in vivo*. This confirms that the repressor activity of E8^E2 is required for tumor formation and genetically defines a conserved E8 interaction domain. E8^E2 prevents expression of E4 protein in basal-like, undifferentiated keratinocytes and thereby their arrest in G2 phase. Since binding of E8^E2 to NCoR/SMRT-HDAC3 co-repressor is required to enable expansion of infected cells in the basal layer and wart formation *in vivo*, this interaction represents a novel, conserved, and potentially druggable target.

## INTRODUCTION

Over a dozen types of human papillomaviruses (HPV), classified as high risk (HR), cause 630,000 new cancer cases of the cervix, head and neck, and anogenital region, among others (1). Effective prophylactic vaccines are available against several HR HPV types, but they do not have therapeutic effects, suggesting a need for therapeutic reagents that could broadly target HR-HPV infection and pre-invasive lesions (2). This could be particularly beneficial given the widespread use of cervical screening by cytology and HR-HPV testing to identify infected individuals. Although generally benign, better treatments for cutaneous HPV diseases are needed, especially in immunocompromised individuals.

HPV have coupled their replication cycle to the differentiation state of the infected keratinocyte. In keratinocytes of the epithelial basal layer, only limited viral replication for genome maintenance takes place (~10–100 episomes/cell), and only early viral genes E1, E2, E5, E6, E7 and their splice variants are expressed. Upon cell division, infected cells can stay in the basal layer or enter the suprabasal layer. Infected cells in the suprabasal spinous layer express S-phase markers and activate cellular DNA replication (3). In the spinous and granular layers of the epithelium, infected cells then activate the differentiation-dependent viral late promoter located in *E7*, undergo vegetative replication to profoundly amplify the genome copy number/cell, and begin to express the E4 protein. Thus, despite its name, E4 is effectively a non-structural late protein. E4 is expressed from the spliced *E1^E4* mRNA and localizes in the cytoplasm (3, 4). E4 has been suggested to contribute to viral genome amplification, the induction of a G2 arrest, the modulation of kinase activity, and virus assembly and release (4). The viral late promoter is also responsible for the expression of the L1 and L2 capsid proteins, which occurs in the uppermost layers of the epithelium. Despite intensive research, the mechanisms controlling the switch to the productive phase remain ill-defined.

Genome replication of HPV is activated by the viral E1 and E2 proteins (5, 6). E1 and E2 are sequence-specific DNA-binding proteins acting as a complex to recognize the origin in the upstream regulatory region (URR) and activate viral replication (5, 6). E2 also has transcription-modulating activities and is involved in the nuclear retention of viral genomes upon cell division by binding mitotic chromosomes (6). Full-length E2 consists of a conserved N-terminal domain of approximately 200 aa that activates DNA replication via the interaction with E1 and is important for transcription modulation and the nuclear retention function (6). The conserved C-terminal domain of approximately 80 aa mediates sequence-specific DNA binding and the dimerization of E2 proteins (6). Both domains are linked by a non-conserved, unstructured “hinge” region of approximately 80 aa (6). In addition to full-length E2, PV express a spliced transcript encoding an E8^E2 fusion protein (7). The *E8^E2* transcript is processed at a splice donor in the *E1* gene and the major splice acceptor in the *E2/E4* region (7). The E8^E2 protein consists of the short E8 domain (~10 aa) which is linked to the E2 hinge-DNA binding/dimerization domain (7). E8^E2 interacts with E2-binding sites (E2BS) and acts as a repressor of viral transcription and genome replication (7). Repression activity by E8^E2 requires not only the specific DNA recognition domain of E2 but also E8 to interact with host cell proteins. Notably, a conserved interaction between E8^E2 and the cellular NCoR (nuclear receptor corepressor), SMRT (silencing mediator of retinoic acid and thyroid hormone receptor), HDAC3 (histone deacetylase 3), GPS2 (G protein pathway suppressor 2), TBL1 (transducin beta-like protein 1), and TBLR1 (transducin beta-like protein 1-related protein) proteins, which assemble into NCoR/SMRT complexes, takes place that has been implicated in mediating both transcription and DNA replication repression activities (8 - 10). NCoR/SMRT complexes mainly repress transcription when targeted to gene loci by an interaction with different DNA-binding transcription factors (11, 12). However, it is unclear whether this functional interaction is required for wart formation.

The disruption of E8^E2 expression in different HPV genomes by mutating the only *E8* start codon, introducing translation termination linkers in *E8*, or inactivating the *E8* splice donor site results in increased viral gene expression and genome replication in short-term assays (13 - 19). An identical phenotype has been observed with HPV16 or 31 genomes which express E8^E2 proteins incapable of interacting with NCoR/SMRT complexes (18, 20). Interestingly, long-term assays with stable cell lines have revealed that HPV16 E8- genomes can be maintained at elevated copy numbers as extrachromosomal elements, whereas HPV31 E8- genomes integrate into the host chromosomes (14, 18, 19). However, analyses of HPV16 have indicated that the loss of E8^E2 enhances viral late protein expression in differentiated cells (18). Recent studies with HPV from the genus beta have revealed that E8^E2 strongly represses E6 and E7 transcription which, in the case of HPV49, prevents immortalization of normal human keratinocytes (15, 16). These observations indicate that E8^E2 is a crucial regulator of viral genome maintenance, productive replication, and immortalization in a virus-type dependent manner but do not address its role in wart production and completing the entire virus life cycle.

Papillomaviruses are, with very few exceptions, highly species-specific; thus, the phenotypes of HPV mutant genomes cannot be addressed *in vivo*, and studies with organotypic cultures cannot address all aspects of the virus life cycle. The mus musculus papillomavirus 1 (MmuPV1) belonging to the genus pi is a useful model, as it allows analyses of viral mutants on tumor formation in laboratory mice (21). The overall replication strategy of MmuPV1 resembles the differentiation-dependent life cycle of HPV as it induces epithelial lesions, and the viral late proteins E4 and L1 are expressed in suprabasal layers of such lesions (22 - 25). MmuPV1 encodes and transcribes E8^E2, and we recently reported that the mutation of the E8 start codon (E8-) in the MmuPV1 genome resulted in greatly increased viral gene expression in cultured normal mouse tail keratinocytes (NMTKs) (26). In line with this, the MmuPV1 (m) E8^E2 protein inhibits MmuPV1 promoter activity and the E1- and E2-dependent activation of the viral origin of replication indicating that MmuPV1 is a suitable model to address the relevance of E8^E2 *in vivo* (26).

Surprisingly, MmuPV1 E8- genomes did not induce warts in T cell-deficient FoxN1^nu/nu^ mice suggesting that E8^E2 is necessary for tumor formation *in vivo* (26). This unexpected phenotype raised the concern whether the E8- phenotype *in vivo* is caused only by a loss of E8^E2 expression or also by *cis*-acting sequences overlapping with the *E8* start codon. We have, therefore, now analyzed additional mutations in MmuPV1 *E8* that disrupt splicing of the *E8^E2* transcript or introduce a translation termination linker in *E8*. Furthermore, we provide evidence that mE8^E2 uses NCoR/SMRT complexes for its repressive activities comparable to its HPV counterparts. We find that MmuPV1 genomes predicted to disrupt E8^E2 expression or activity display increased viral gene expression in cultured murine keratinocytes but are unable to induce wart formation in T cell-deficient mice confirming that E8^E2 is required for this. We now also provide evidence that E8^E2 limits expression of the late viral E4 protein in mouse keratinocytes maintained in monolayer culture. Consistent with the data reported for HPV E4 proteins (27 - 29), MmuPV1 E4-positive murine keratinocytes show an increase in cells in the G2/M and a decrease in the cells in the S-phase of the cell cycle. This strongly suggests that the loss of E8^E2 induces the late, vegetative phase of the viral replication and a shift toward the G2 cell cycle phase in undifferentiated cells. This most likely prevents cell division of infected cells *in vivo* which consequently prevents tumor formation *in vivo*.

## RESULTS

### Disruption of the *E8* splice donor increases MmuPV1 gene expression in murine keratinocytes

**Fig 1:**
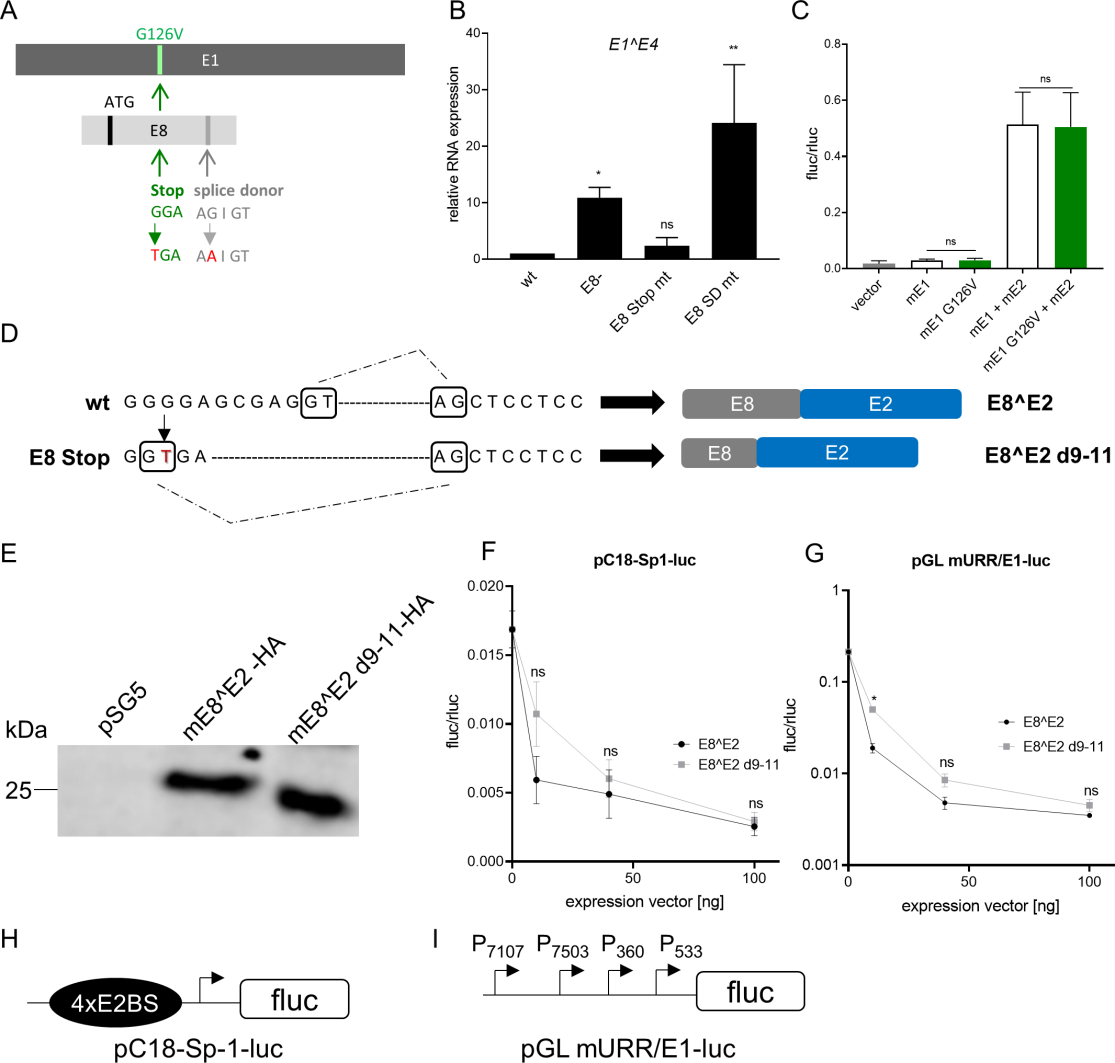
The MmuPV1 E8 Stop mutant genome creates a new splice donor site resulting in E8^E2 d9-11. (**A**) Schematic representation of the *E8* ORF (light gray) and the overlapping *E1* reading frame (dark gray). The ATG start codon in *E8* and the splice donor sequence are indicated by black and gray lines in E8, respectively. The E8 Stop mt results in a G126 to V exchange in E1 (in light green), whereas the E8 splice donor mt is silent in E1. (**B**) NMTKs were transfected with re-circularized MmuPV1 genomes as indicated. RNA was harvested 6 d p. t. and analyzed by qPCR for *E1^E4* expression relative to *PGK1*. Values represent the average from three to four independent experiments and are relative to the wt (=1). Error bars indicate the standard error of the mean (SEM), and statistical significance was determined by a one-way ANOVA test (ns, not significant; *P < 0.05; **P < 0.01). (**C**) C33A cells were transfected as indicated with 100 ng of pGL mURR/E1-luc, 10 ng pCI-neo-Rluc, 1,000 ng pSG mE1 or pSG mE1 G126V, 100 ng pSG mE2, or empty vector (pSG5) to obtain equal amounts of plasmid DNA. Luciferase activities were determined 48 h p.t. Data are presented as ratios between firefly luciferase (fluc) and renilla luciferase (rluc) activities. Averages are derived from three independent experiments, and the error bars represent the SEM. Statistical significance was determined by unpaired *t*-test (ns, not significant). (**D**) The G to T mutation in MmuPV1 E8 Stop mt creates a new splice donor site resulting in E8^E2 d9-11. Intron nucleotides determining splice donor and acceptor sequences are framed. Dashed lines indicate splice junctions. (**E**) Western blot of extracts from C33A cells transfected with pSG5, mE8^E2-HA wt, or mE8^E2 d9-11-HA using an anti-HA antibody. (**F**) C33A cells were transfected with 100 ng of pC18-Sp1-luc, 10 ng pCI-neo-Rluc, and the indicated amounts of pSG mE8^E2-HA wt or d9-11, or empty vector (pSG5), to obtain equal amounts of plasmid DNA. Luciferase activities were determined 48 h p.t. Data are presented as ratios between fluc and rluc activities. Averages are derived from three independent experiments, and the error bars represent the SEM. Statistical significance was determined by one-way ANOVA test (ns, not significant). (**G**) C33A cells were transfected with 100 ng of pGL mURR/E1-luc reporter plasmid, 10 ng pCI-neo-Rluc, 1,000 ng pSG mE1, 100 ng pSG mE2, and the indicated amounts of pSG mE8^E2-HA wt or d9-11, plus empty vector (pSG5), to obtain equal amounts of plasmid DNA. Luciferase activities were determined 48 h p.t. Data are presented as ratios between firefly luciferase (fluc) and renilla luciferase (rluc) activities. Averages are derived from at least three independent experiments, and the error bars represent the SEM. Statistical significance was determined by one-way ANOVA test (ns, not significant; *P < 0.05). (**H**) Schematic structure of the pC18-SP1-luc plasmid containing four E2 binding sites (E2BS). (**I**) Schematic structure of the pGL mURR/E1-luc plasmid. Transcription start sites of the P7107, P7503, P360, and P533 promoters are indicated.

To provide further evidence that the lack of wart formation *in vivo* by the *E8-* genome is due to the lack of E8^E2 rather than *cis*-elements overlapping with the E8 start codon, we generated additional mutations in *E8* predicted to prevent E8^E2 expression by introducing a stop codon in *E8* (G9X; E8 Stop mt) or disrupting the splice donor signal AG|GT (AA|GT; E8 SD mt) (Fig. 1A). WT, E8-, E8 Stop mt, or E8 SD mt genomes were transfected into NMTK, and the amount of *E1^E4* transcripts was measured by qPCR 6 d post transfection (p.t.) (Fig. 1B). E8 SD mt increased *E1^E4* transcripts to significantly higher levels than the wt, and to slightly higher levels than E8- genomes, consistent with the idea that the spliced *E8^E2* RNA produces the E8^E2 repressor protein. Surprisingly, expression from E8 Stop mt genomes was only slightly increased, and this was not significant. In contrast to E8- and E8 SD mt genomes, the *E1* coding sequence in E8 Stop mt genomes is affected such that E1 G126 is exchanged to V (Fig. 1A). However, reporter assays using the pGL mURR/E1-luc plasmid, which harbors several MmuPV1 promoters and the viral origin of replication, revealed similar activation levels for WT mE1 and mE1 G126V in the presence of mE2 (Fig. 1C and H). This suggested that the lack of a transcriptional phenotype of E8 Stop mt is not due to changes in E1. When evaluating the sequences surrounding the E8 Stop mt, we noticed that the change from G to T created a potential novel splice donor sequence (CGGTGA, Fig. 1D). To investigate this in more detail, reverse transcriptase-PCR was performed using RNA isolated from WT or E8 Stop mt transfected NMTK and primers in the E8 and E4 region. DNA sequencing of gel-purified amplicons revealed that, as expected, WT-transfected cells express the E8^E2 transcript spliced from nt. 1125 to 3139 (Fig. 1D). In contrast, in E8 Stop mt-transfected cells, nt. 1116 was linked to nt. 3139 providing evidence that the introduction of the stop codon accidentally created a novel splice donor sequence (Fig. 1D). Use of the splice donor at nt. 1116 not only removes the artificial stop codon at codon 9 of E8 but also creates an in-frame fusion of E8^E2 which only misses residues 9–11 (mE8^E2 d9-11, Fig. 1D). Immunoblot analysis indicated that HA-tagged mE8^E2 d9-11 is expressed at similar levels as mE8^E2 in C33A cells from transfected expression vectors (Fig. 1E). Transient reporter assays in C33A cells using the pGL mURR/E1-luc reporter co-transfected with mE1 and mE2 and different amounts of mE8^E2 or mE8^E2 d9-11 expression vectors revealed that E8^E2 d9-11 has repressive activity comparable to wt E8^E2 (Fig. 1G and I). Similar results were obtained with an E2BS reporter without mE1 and mE2 (pC18-Sp1-luc, Fig. 1F and H). These data strongly suggest that the lack of increased gene expression from E8 Stop mt genomes is most likely due to the expression of E8^E2 d9-11 which compensates for wt E8^E2.

### Interaction of NCoR/SMRT co-repressor components with mE8^E2 contributes to transcriptional repression

**Fig 2:**
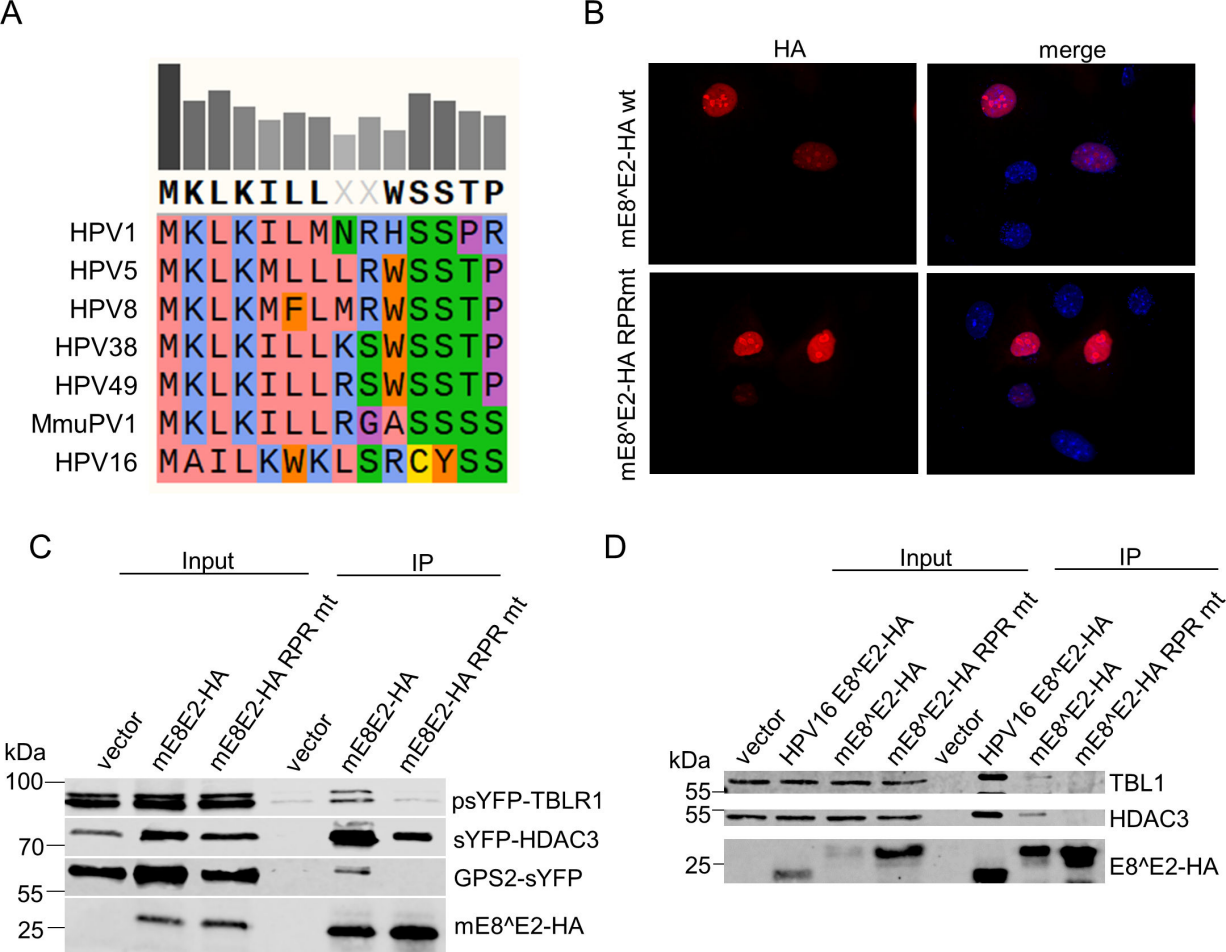
MmuPV1 E8^E2 interacts with different NCoR/SMRT complex components. (**A**) The E8 consensus sequence of different PV reveals a highly conserved KLK motif. E8 sequences were retrieved from the PAVE database (31) and analyzed with Snapgene (version 5.0.8). (**B**) NMTKs were transfected with mE8^E2-HA wt or RPR mt expression plasmids, fixed, and stained with anti-HA antibodies and DAPI 48 h p.t. (**C**) Co-immunoprecipitation analysis reveals an interaction of wt MmuPV1 E8^E2-HA with transfected sYFP-GPS2, sYFP-TBLR1, or sYFP-HDAC3, which is decreased/abolished with mE8^E2-HA RPR mt. Cell lysates from C33A cells were directly analyzed (input) or precipitated with α-HA antibody (IP) and subjected to immunoblot analysis with anti-sYFP and anti-HA antibodies. (**D**) Co-immunoprecipitation analysis reveals an interaction of HPV16 E8^E2-HA and mE8^E2-HA with endogenous HDAC3 and TBL1. HeLa cells were transfected with the empty vector or expression plasmids for HPV16 E8^E2-HA, mE8^E2-HA, and mE8^E2-HA RPR mt, and cell lysates were directly analyzed (input) or precipitated with α-HA antibody (IP) and subjected to immunoblot analysis with the indicated antibodies.

HPV1, 8, 16, and 31 E8^E2 proteins have been shown to functionally interact with NCoR/SMRT co-repressor complexes that consist of NCoR and/or SMRT, HDAC3, TBL1 and/or TBLR1, and GPS2 (9 - 12). This interaction requires conserved K5/W6/K7 residues in HPV16 and 31 E8 or K2/L3/K4 residues in HPV8 E8 (9). A sequence alignment of HPV1, 5, 8, 38, 49, and MmuPV1 E8 revealed that MmuPV1 harbors conserved K2/L3/K4 residues pointing to the possibility that MmuPV1 also functionally interacts with NCoR/SMRT complexes (Fig. 2A). To test this, HA-tagged mE8^E2 was co-expressed with GPS2, HDAC3, or TBLR1 fused to sYFP and analyzed by co-immunoprecipitation (co-IP) analysis in C33A cells. In addition, conserved K2/L3/L4 residues were exchanged to RPR in mE8^E2 (mE8^E2 RPR mt). Immunoblot and immunofluorescence analysis revealed that mE8^E2 RPR mt is stably expressed and localizes to the nucleus as for the wt protein (Fig. 2B through D). Co-IP analyses revealed that mE8^E2 interacts with HDAC3, GPS2, and TBLR1, whereas mE8^E2 RPR mt, despite being expressed at similar levels in the nucleus, was greatly impaired (Fig. 2C). Attempts to express full-length NCoR as a sYFP fusion protein to analyze the interaction with mE8^E2 failed for unknown reasons. However, since HDAC3 and GPS2 interact with mE8^E2, which are known to directly bind to NCoR/SMRT but not directly to each other (30), it can be assumed that NCoR/SMRT complexes interact with mE8^E2. In line with this, the endogenous NCoR/SMRT complex components HDAC3 and TBL1 can be immunoprecipitated with HPV16 E8^E2-HA and mE8^E2-HA but not with the mE8^E2-HA RPR mt in HeLa cells transfected with the respective expression vectors (Fig. 2D). We noted that HPV16 E8^E2 interacts more efficiently with HDAC3 and TBL1 than mE8^E2 which could either be due to species-specific differences in NCoR/SMRT components or differences in the E8 domain (Fig. 2A).

**Fig 3:**
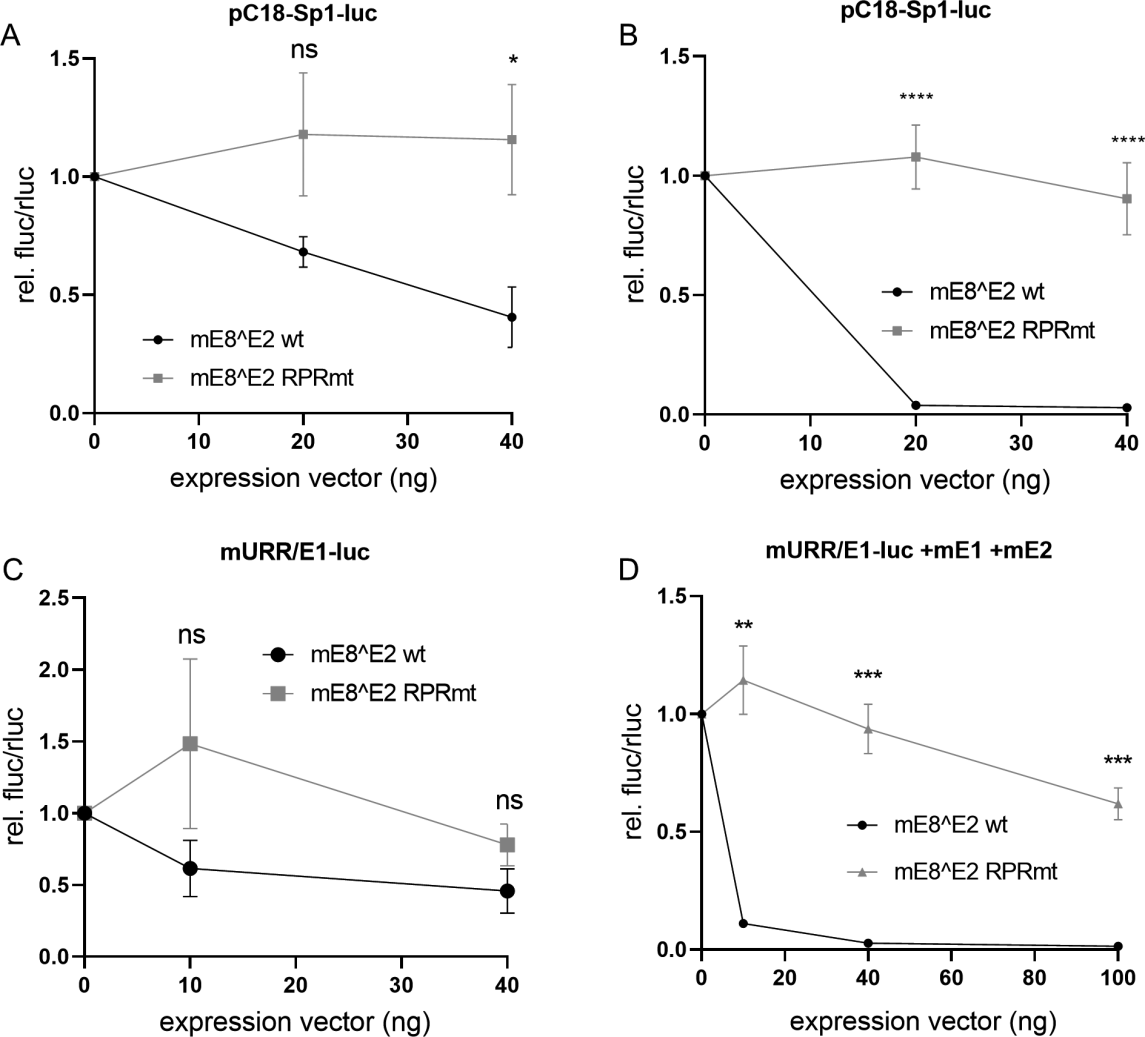
Lack of repression by mE8^E2 correlates with binding to NCoR/SMRT complexes. (**A**) C33A cells or (**B**) NMTKs were transfected with 100 ng of pC18-Sp1-luc reporter plasmid, 10 ng pCI-neo-Rluc, and the indicated amounts of pSG mE8^E2-HA or RPR mt, plus empty vector (pSG5), to obtain equal amounts of plasmid DNA. Luciferase activities were determined 48 h p.t. Data are presented as ratios between fluc and rluc activities relative to those of pSG5-transfected cells. Averages are derived from three independent experiments, and error bars represent the SEM. Statistical significance was determined by an unpaired *t*-test (ns, not significant; *P < 0.05; ***P < 0.001). (**C and D**) mE8^E2 inhibits MmuPV1 promoter activity in the presence of mE1 and mE2. C33A cells were transfected with 100 ng of pGL mURR/E1-luc reporter plasmid, 10 ng pCI-neo-Rluc, and the indicated amounts of pSG mE8^E2-HA or RPR mt, empty vector (pSG5), to obtain equal amounts of plasmid DNA and in (**D**) additionally 1,000 ng pSG mE1 and 100 ng pSG mE2. Luciferase activities were determined 48 h p.t. Data are presented as ratios between fluc and rluc activities relative to those of pSG5-transfected cells. Averages are derived from three independent experiments, and error bars represent the SEM. Statistical significance was determined by an unpaired *t*-test (ns, not significant; **P < 0.01; ***P < 0.001).

To evaluate, if the interaction of mE8^E2 with NCoR/SMRT components is important for repression activity, reporter assays were conducted. Whereas WT mE8^E2 represses pC18-Sp1-luc activity in both human C33A cells and NMTK, mE8^E2 RPR mt has no inhibitory activity (Fig. 3A and B). Furthermore, the pGL mURR/E1 construct is repressed by low amounts of mE8^E2 expression vector, whereas E8^E2 RPR mt even slightly activates the reporter at low input although these differences were not statistically significant (Fig. 3C). When adding mE1 and mE2 to pGL mURR/E1-luc to induce replication, the differences between mE8^E2 and mE8^E2 RPR mt are even more pronounced: whereas E8^E2 represses the reporter almost completely at 10 ng input, mE8^E2 RPR mt only slightly inhibits at 100 ng input (Fig. 3D). These data strongly suggest that the conserved KLK residues not only are important for the interaction with NCoR/SMRT complex components but also for repression activity in both human and murine cells.

**Fig 4:**
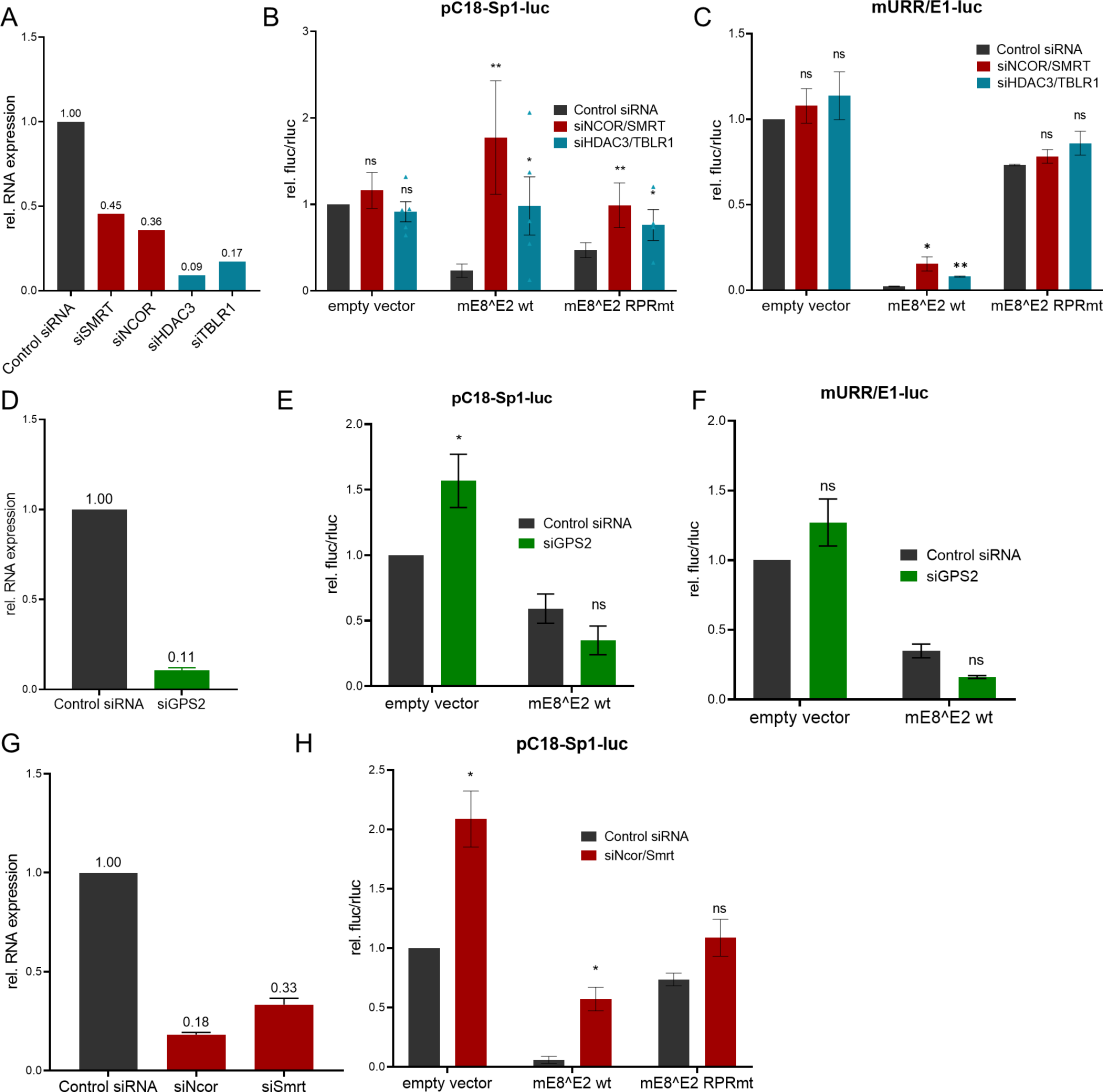
NCoR/SMRT complex mediates repression of transcription and replication by mE8^E2 in C33A and NMTK. (**A**) Knockdown efficiency of siRNAs targeting SMRT, NCoR1, HDAC3, and TBLR1 in C33A cells compared to control siRNA determined by qPCR using PGK1 as a reference gene and control siRNA. (B and C) siRNA against NCoR/SMRT and HDAC3/TBLR1 relieves the repression of transcription (**B**) and replication (**C**) by mE8^E2. C33A cells were transfected with siRNA combinations and 24 h later with the pC18-Sp1-luc reporter (**B**) or mURR/E1-luc (**C**) as well as the expression vectors for mE8^E2 WT or RPR mt. Luciferase activities were determined 48 h p.t. Averages are derived from at least three independent experiments, and error bars represent the SEM. (**D**) Knockdown efficiency of siRNAs targeting *GPS2* in C33A cells compared to control siRNA. (E and F) siRNA against GPS2 does not relieve the repression of transcription (**E**) and replication (**F**) C33A cells were transfected with siGPS2 and 24 h later with the pC18-Sp1-luc (**E**) or mURR/E1-luc (**F**) as well as the expression vectors for mE8^E2 WT Luciferase activities were determined 48 h p.t. Averages are derived from at least three independent experiments and are expressed relative to empty vector/control siRNA. (**G**) Knockdown efficiency of siRNAs for *Smrt* and *Ncor1* in NMTK compared to control siRNA determined by qPCR using *PGK1* as a reference gene. (**H**) siRNAs targeting Ncor1/Smrt relieve the repression of transcription by mE8^E2 in NMTK. NMTKs were transfected with siRNAs for Ncor1 and Smrt and 24 h later with the pC18-Sp1-luc reporter as well as the expression vectors for mE8^E2 WT or RPR mt. Luciferase activities were determined 48 h p.t. Averages fare derived from four independent experiments, and error bars indicate the SEM. Statistical significance was determined by a ratio paired *t*-test (ns, not significant; *P < 0.05; **P < 0.01).

To further confirm, that repression is mediated by NCoR/SMRT complexes as suggested by co-IP experiments, RNA interference experiments were carried out. Previous studies have indicated that combinations of siRNAs against different complex components are necessary to attenuate repression activity of E8^E2, which is most likely due to the functional redundancies of NCoR and SMRT and TBL1 and TBLR1 proteins and/or incomplete target mRNA loss (9, 30). We, therefore, tested if combinations of siNCoR and siSMRT or of siHDAC3 and siTBLR1 are required for repression by mE8^E2. In addition, siGPS2 was included which was previously not tested. All siRNAs significantly reduced gene expression of the respective target genes in human or mouse cells (Fig. 4A, D, and G). SiNCoR/siSMRT had no effect on the reporter itself but greatly relieved repression by mE8^E2, whereas a much smaller effect was seen for mE8^E2 RPR mt on pC18-Sp1-luc in C33A cells (Fig. 4B). Similarly, siHDAC3/siTBLR1 relieved repression by mE8^E2 but not by mE8^E2 RPR mt. Repression of the pGL-mURR/E1-luc reporter in the presence of mE1 and mE2 by mE8^E2 is also significantly attenuated by both siNCoR/siSMRT and siHDAC3/siTBLR1, whereas no effect on mE8^E2 RPR mt is seen (Fig. 4C). The differences between siRNA knockdown and the E8^E2 RPR mt are most likely due to the remaining levels of NCoR and SMRT (Fig. 4A). A similar, specific effect of siNcor1/siSmrt on the repression of pC18-Sp1-luc by mE8^E2 could also be observed in NMTK (Fig. 4H). In contrast, the knockdown of Gps2, despite being more effective than the NcoR1 and Smrt knockdowns, had no effect on the repression by mE8^E2 of pC18-Sp1-luc or pGL-mURR/E1-luc in the presence of E1 and E2 (Fig. 4D through F). Taken together, these reporter experiments strongly indicate that NCoR/SMRT complexes contribute to the repression activity of mE8^E2.

### NCoR/SMRT components contribute to the inhibition of MmuPV1 gene expression in an E8^E2-dependent manner

**Fig 5:**
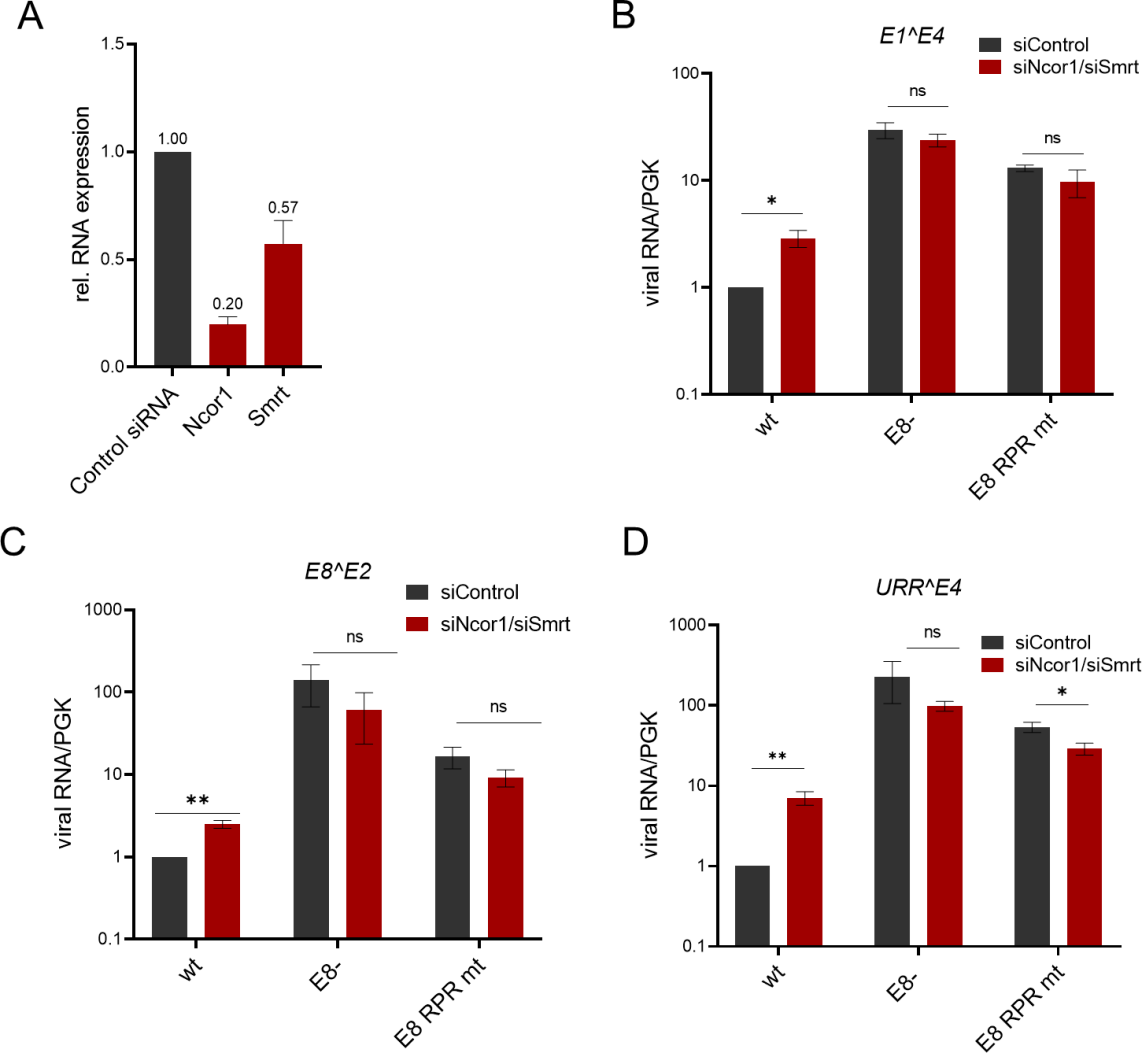
Increased expression of spliced viral transcripts from MmuPV1 E8- and E8 RPR mt genomes in NMTK. (**A**) Knockdown efficiency of siRNAs targeting *Ncor1* and *Smrt* in mouse keratinocytes compared to control siRNA determined by qPCR using *PGK1* as a reference. (**B–D**) Primary mouse keratinocytes were transfected with MmuPV1 genomes as indicated and transfected with control siRNA or siNcor/siSmrt the next day. RNA was harvested 5 d p.t., and the expression of spliced MmuPV1 *E1^E4*, *E8^E2,* or *URR^E4* transcripts was determined by qPCR using *PGK1* as a reference gene. Expression is shown relative to wt genomes in the presence of control siRNA. Averages are derived from four independent experiments, and error bars indicate the SEM. Statistical significance was determined by an unpaired *t*-test (ns, not significant; *P < 0.05; **P < 0.01).

We next evaluated the contribution of NCoR/SMRT complexes to gene expression from MmuPV1 WT, E8-, or E8 RPR mt genomes in NMTK. The exchange of E8 KLK to RPR is silent in the overlapping *E1* gene, and therefore, phenotypes should be only due to E8^E2. In the presence of control siRNA, E8 RPR mt genomes displayed higher levels of *E1^E4* (13-fold), *E8^E2* (16-fold), and *URR^E4* (54-fold) transcripts than WT genomes, consistent with a loss of repression activity by mE8^E2 (Fig. 5). The simultaneous knockdown of *Ncor1* and *Smrt* by siRNA significantly increased *E1^E4* (2.9-fold), *E8^E2* (2.5-fold), and *URR^E4* (7.1-fold) transcripts from WT genomes but reduced transcription from E8- and E8 RPR mt genomes. These data are consistent with the idea that mE8^E2 interacts with NCoR/SMRT complexes to limit viral gene expression in primary keratinocytes.

### Disruption of E8^E2 activity prevents tumor formation by MmuPV1 genomes in athymic nude *FoxN1*^nu/nu^ mice

**Fig 6:**
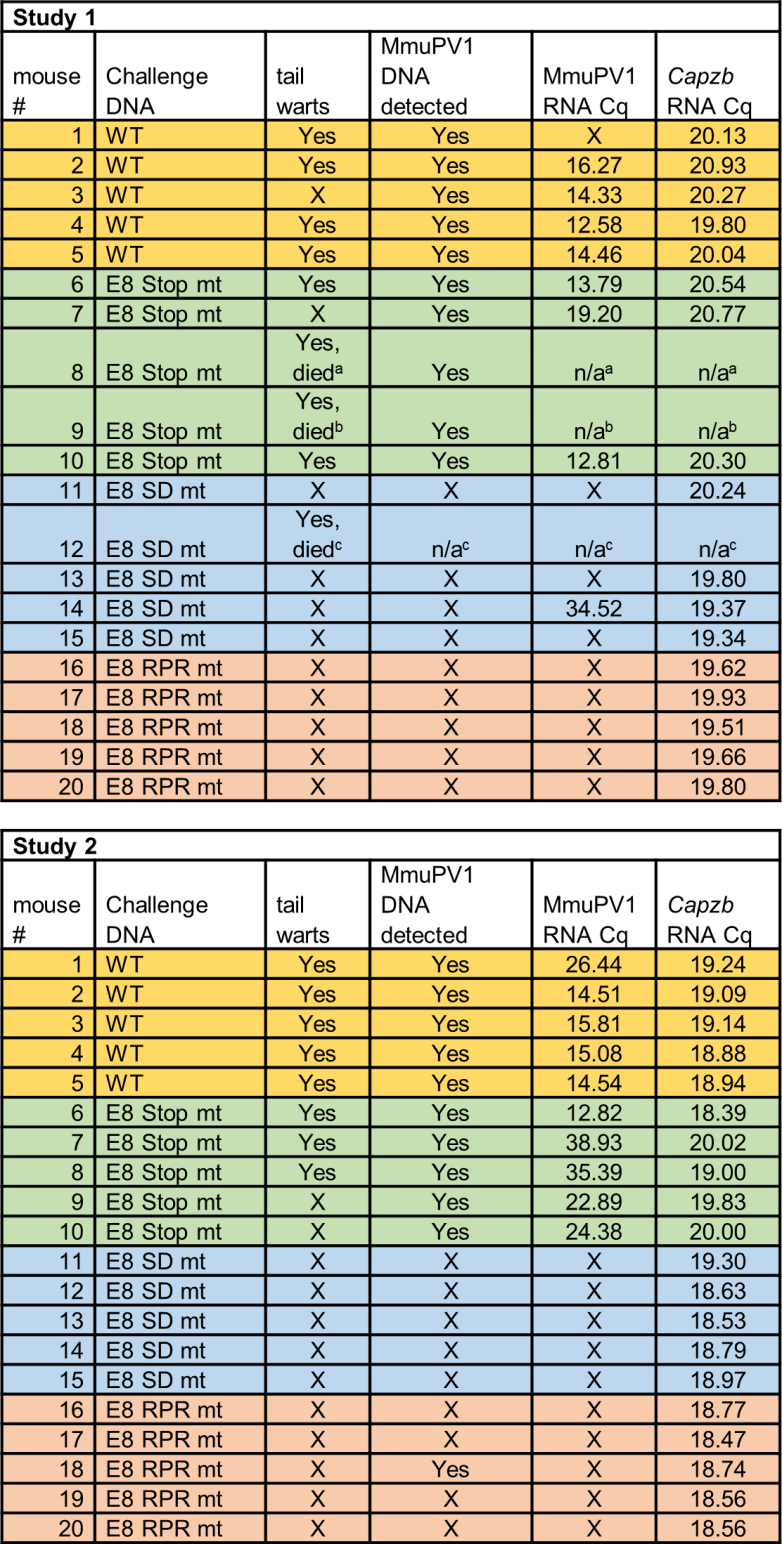
Disruption of E8^E2 binding to co-repressor by defined mutants prevents tumor formation by MmuPV1 genomic DNA in athymic nude *Foxn1*^nu/nu^ mice. Wild-type and mutant MmuPV1 genomic DNA was introduced in the tail skin of five *Foxn1*^nu/nu^ mice per genome in two different studies. All mice were euthanized and examined for tail warts (at 4 or 5 months after DNA challenge in studies 1 and 2, respectively), except animals that died at ^a^3.5 months, ^b^4 months, and ^c^0.25 months post DNA challenge for unrelated reasons. Tail tissues were excised and analyzed for the presence of MmuPV1 DNA and transcripts.

Next, we determined if MmuPV1 E8 Stop mt, E8 SD mt, or E8 RPR mt genomes induce warts in athymic nude *FoxN1*^nu/nu^ mice using the WT genome as a control. MmuPV1 genomes were introduced in two independent experiments in the tail skin of 10 *FoxN1*^nu/nu^ mice per genome and analyzed after 4 and 5 months, respectively. This revealed that the WT induced warts in 9/10 mice and the E8 Stop mt in 7/10 mice (Fig. 6). In contrast, the E8 SD mt and E8 RPR mt genomes failed to induce visible warts (0/9 and 0/10 mice, respectively). The presence of MmuPV1 mRNA in challenge sites with visible lesions was confirmed by qRT-PCR and absent from those without (except for two E8 Stop mt-challenged mice where DNA and RNA were detected but which did not develop warts within the timeframe of this study and one E8 SD mt-challenged mouse at a high Cq where no viral DNA was detected), confirming that they are, indeed, viral warts. This was also confirmed by histologic analyses. Likewise, PCR was utilized to amplify viral DNA from each challenge site sample. Viral sequences amplified from the WT and E8 Stop mt DNA challenge sites but not the E8 SD mt or E8 RPR mt challenges, with one exception (1/10 E8 RPR mt). Nevertheless, this suggests that the detection of MmuPV1 DNA was not carry over from the initial challenge but likely reflected *de novo* replication. Furthermore, sequencing of the PCR products confirmed the presence of the expected sequences, showing that the lesions in the E8 Stop mt and E8 RPR mt genome-challenged mice did not reflect contamination with WT virus. Interestingly, one mouse challenged with WT DNA produced tail warts positive for viral DNA, but no mRNA was detected. We speculate that this may be due to an integration event.

In summary, these data confirm and extend previous findings that MmuPV1 E8- genomes display increased gene expression in tissue culture but do not form warts or are maintained in the skin of athymic nude *FoxN1*^nu/nu^ mice (26). Consistent with the idea that this is an E8^E2-dependent phenotype, E8 SD mt and E8 RPR mt genomes show increased gene expression in tissue culture but do not form warts or maintain viral DNA or viral gene expression at challenge sites. These data strongly suggest that the complete inactivation of E8^E2 or expression of a repression-attenuated E8^E2 protein is incompatible with tumor growth *in vivo* even in the absence of T-cells.

### E8^E2 inhibits E4 expression from MmuPV1 genomes in cultured murine keratinocytes

**Fig 7:**
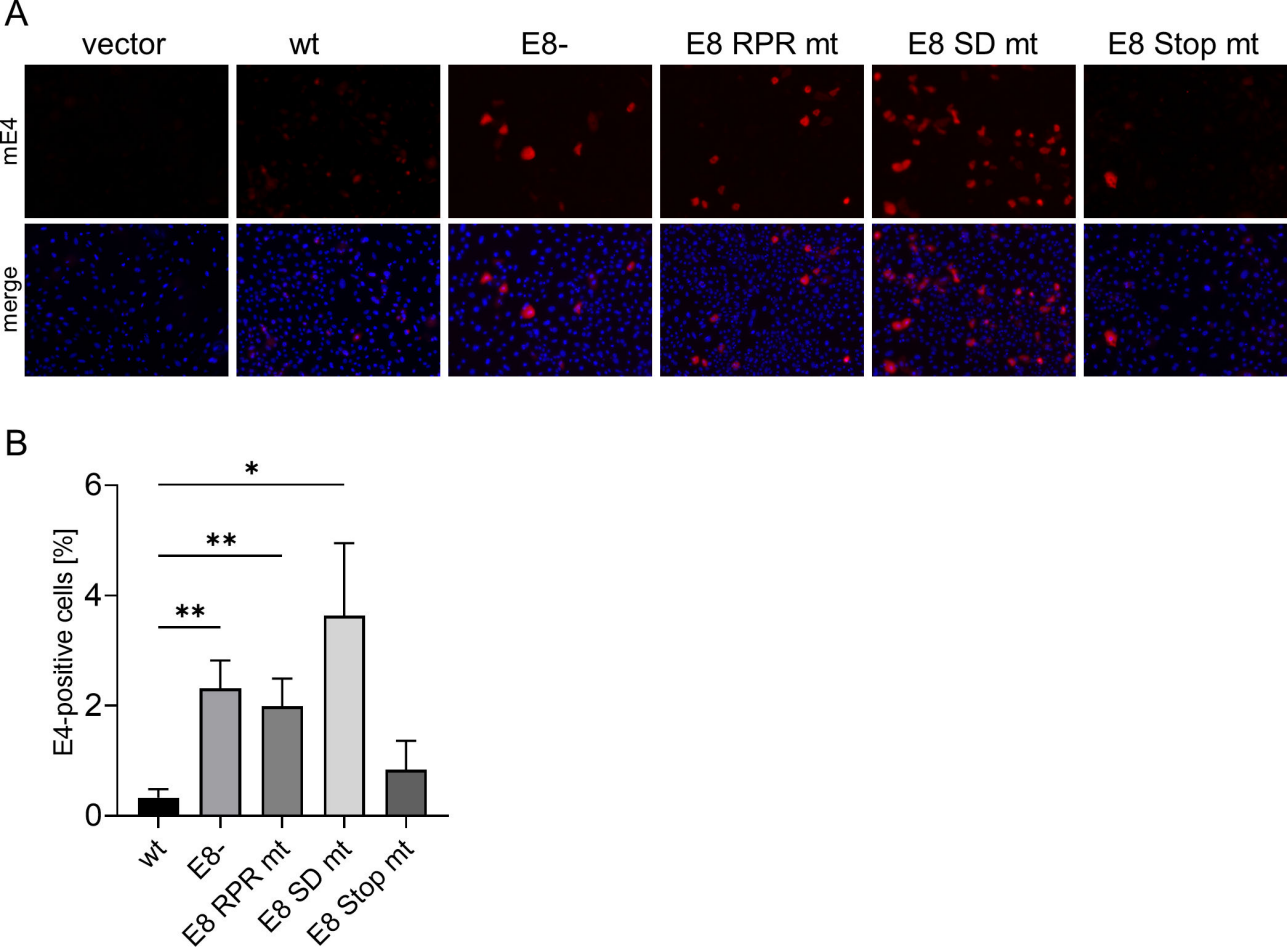
E8^E2 mt genomes increase the numbers of E4-expressing cells. (**A**) NMTKs were transfected with pSG5 (vector), MmuPV1 wt, E8-, E8 RPR mt, E8 SD mt, or E8 Stop mt genomes, fixed 2 d p.t., and stained with mE4 antibody (red) and DAPI (blue). Magnification 100×. (**B**) Flow cytometry analysis of NMTK transfected with MmuPV1 wt or different mt genomes using an anti-mE4 antibody 2 d p.t. Data were analyzed with FlowLogic and the averages are derived from five independent experiments, and error bars indicate the SEM. Statistical significance was determined with one-way ANOVA test and Fisher’s LSD (*P < 0.05; **P < 0.01).

**Fig 8:**
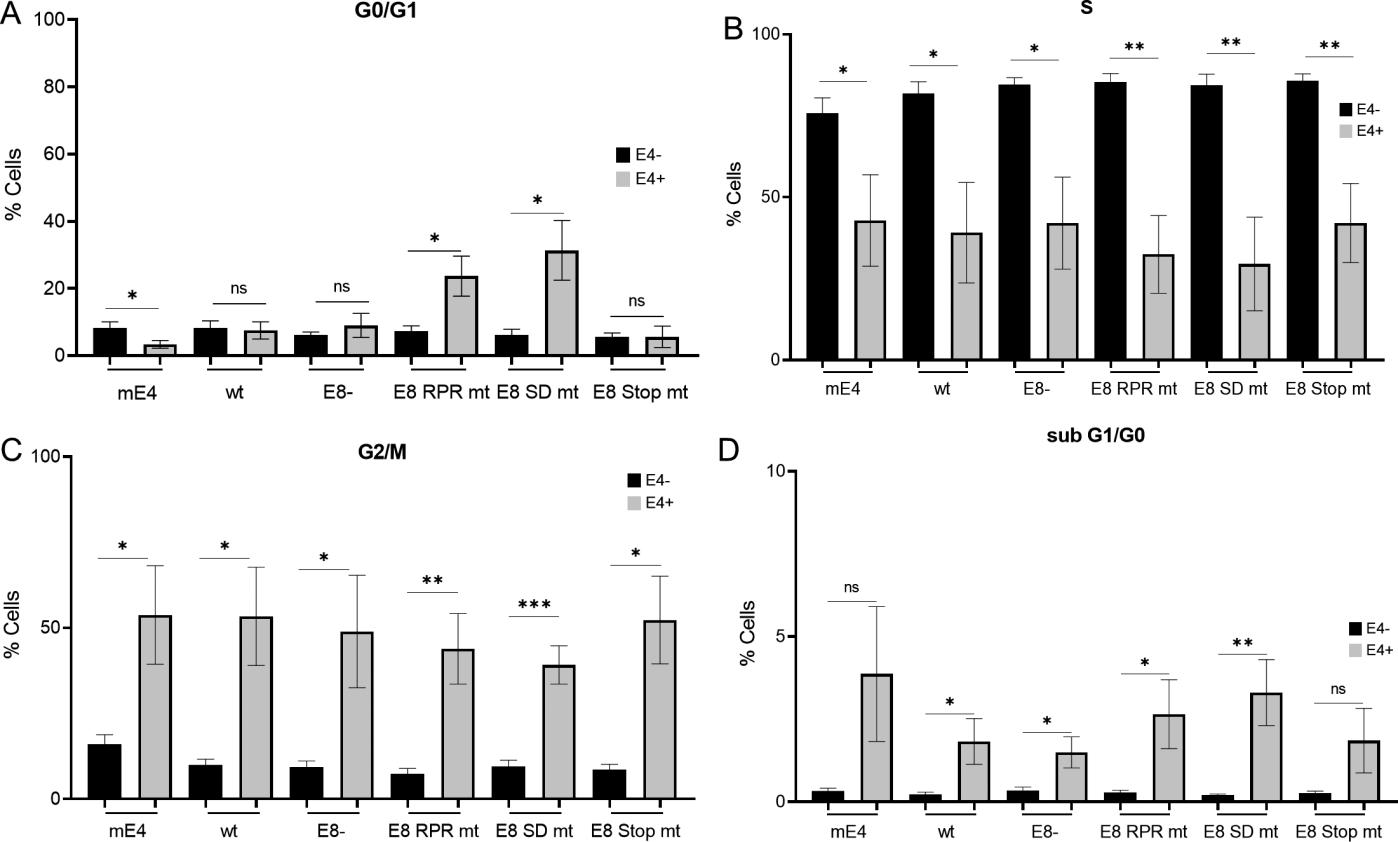
ME4 protein expression changes the cell cycle profile in mouse keratinocytes. Flow cytometry analysis of NMTK transfected with pSG 3xHA-mE4 (mE4) or MmuPV1 WT or different mt genomes using anti-mE4 antibody and propidium iodide to stain DNA. DNA content analysis in E4-negative (E4−) and E4-positive (E4+) cells 2 d p.t. Quantification of E4− and E4+ cell fractions in the G0/G1 (**A**), S (**B**),or G2/M (**C**) phase of the cell cycle using FlowLogic and the Watson Improved analysis. (**D**) Transfected NMTKs were analyzed 5 d p.t. for E4 and DNA content using PI to determine the fraction of apoptotic cells. Data are derived from three independent experiments, and error bars indicate the SEM. Statistical significance was determined with unpaired *t*-test (ns, not significant; *P < 0.05; **P < 0.01; ***P < 0.001).

**Fig 9:**
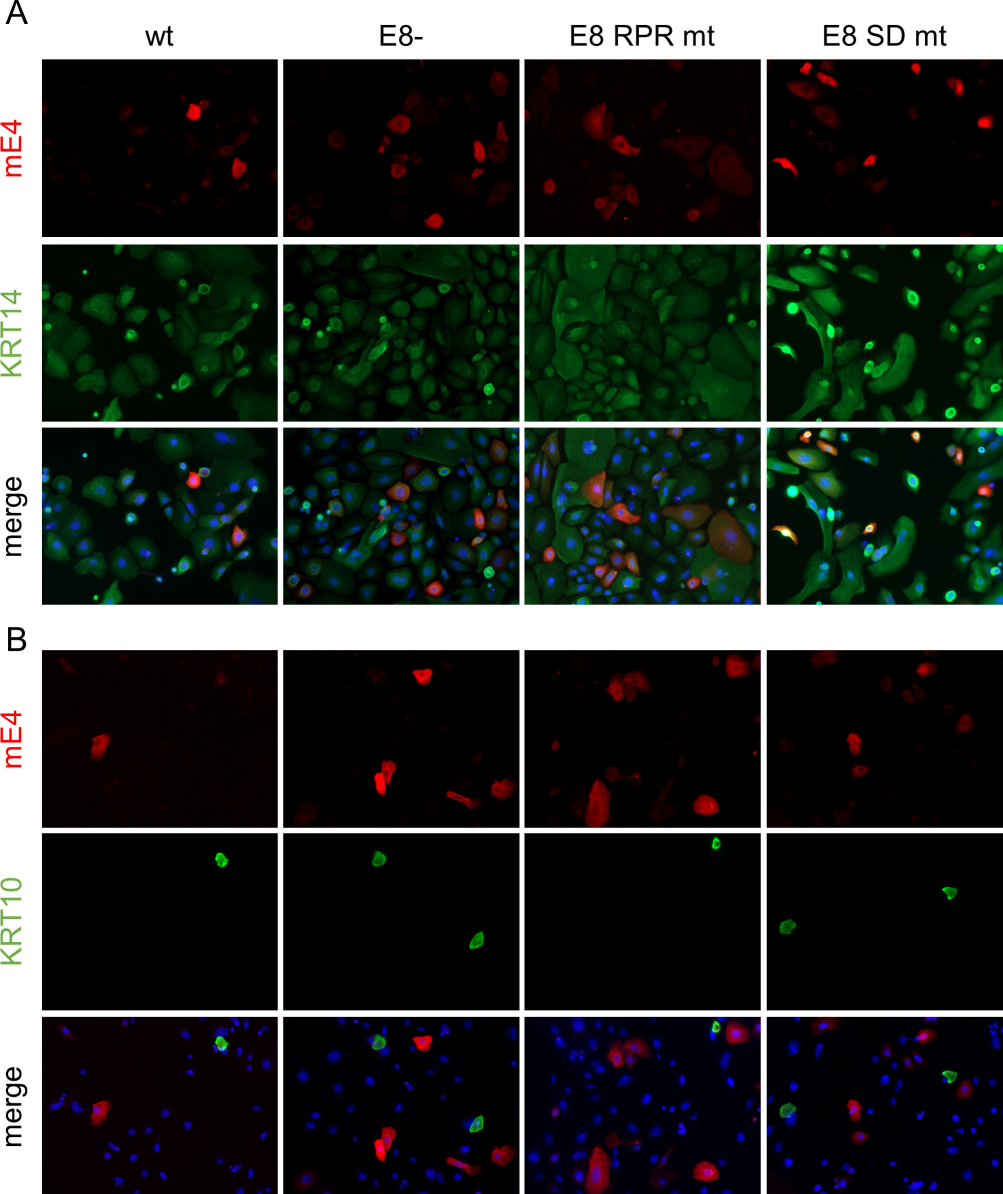
Immunofluorescence staining for mE4 and the keratinocyte differentiation markers keratin 14 and 10. Mouse keratinocytes were transfected with MmuPV1 WT, E8-, E8 RPR mt, E8 SD mt genomes, fixed 2 d p.t., and stained with anti-mE4 (red) and anti-keratin 14 (KRT14, green) or anti-keratin 10 (KRT10, green) antibodies and DAPI to visualize DNA (blue). Magnification 200×.

**Fig 10:**
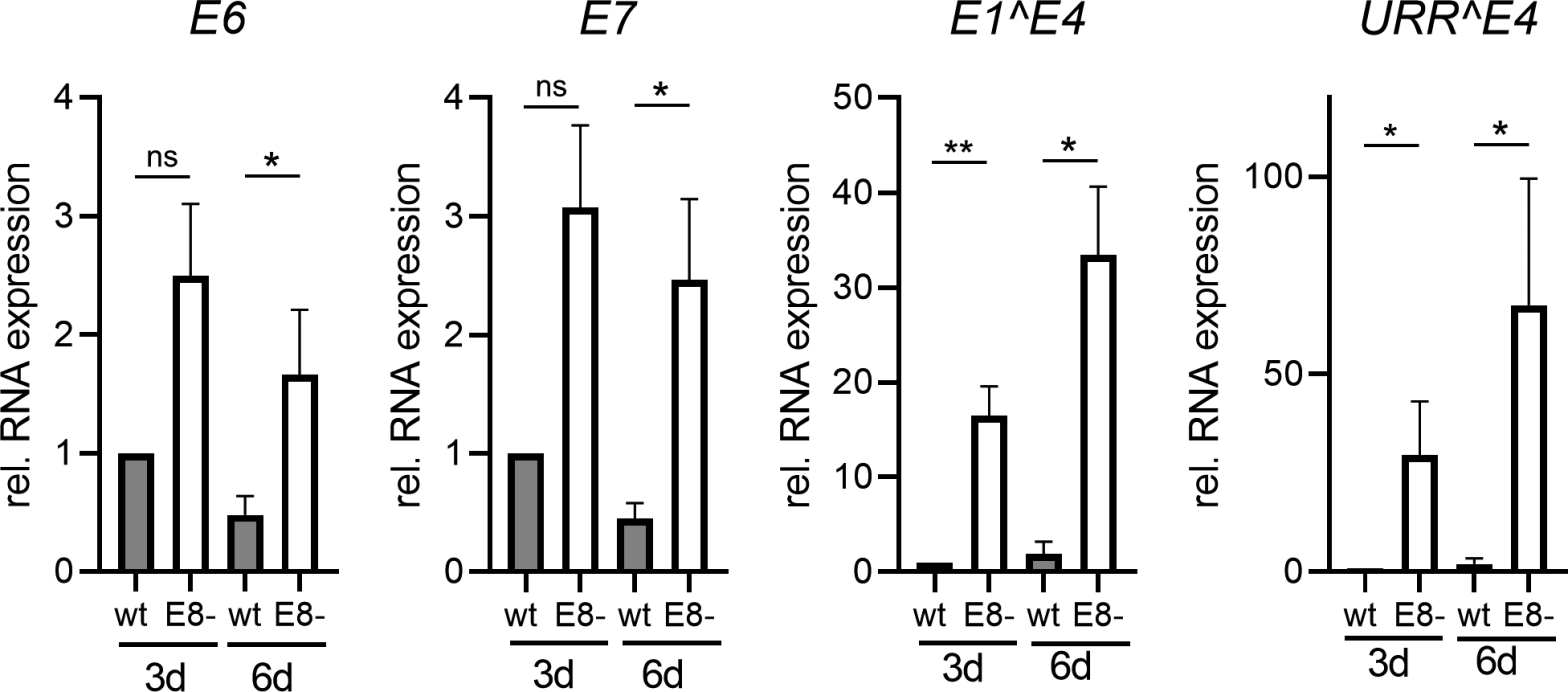
E8- genomes preferentially increase viral late transcripts. NMTKs were transfected with the indicated MmuPV1 genomes, and RNA was harvested 3 d or 6 d p.t. Poly-A+ RNA was analyzed by qPCR to detect *E6*, *E7*, *E1^E4,* or *URR^E4* transcripts and *PGK1* as a reference. Averages are derived from three independent experiments and are presented relative to the wt on d3. Error bars indicate the SEM. Statistical significance was determined by a ratio-paired *t*-test (ns, non-significant; *P < 0.05; **P < 0.01).

The increased genome replication and expression of *E1^E4* transcripts of E8^E2 mt genomes in cultured, undifferentiated NMTK resemble the differentiation-dependent genome amplification of PV characterized by the abundant expression of the viral E4 protein [(26); Fig. 1B and 5B]. We, therefore, speculated that the loss of MmuPV1 E8^E2 induces the switch to productive replication and results in the expression of E4 in NMTK maintained in monolayer culture. We first validated a previously described anti-mE4 polyclonal serum by transfecting an expression vector for HA-tagged MmuPV1 E4 in NMTK and monitored E4 protein expression 2 d p.t. by immunofluorescence analysis (Fig. S1). Transfection of the mE4-3xHA plasmid revealed a colocalization of anti-HA and anti-mE4 signals with an average Pearson correlation coefficient of 0.801 (range: 0.6451–0.950) confirming the specificity of the E4 antiserum. Consistent with data for HPV E4 proteins, mE4 is expressed in the cytoplasm (4). A comparable cytoplasmic staining with anti-mE4 was observed in some MmuPV1 wt and E8- transfected NMTK indicating that mE4 is expressed (Fig. S1). When analyzing NMTK transfected with WT, E8-, E8 Stop mt, E8 SD mt, or E8 RPR mt genomes 2 d p.t., E4-positive cells could be seen with all genomes (Fig. 7A). However, the number of E4-positive cells appeared higher in E8-, E8 SD mt, and E8 RPR mt compared to wt or E8 Stop mt transfected. To quantify this more accurately, flow cytometry experiments were carried out (Fig. 7B). To identify E4-positive cells by flow cytometry, NMTKs were transfected with the empty vector as a negative control and the mE4-3xHA expression vector as a positive control. Consistent with the immunofluorescence experiments, WT (0.3%) and E8 Stop mt (0.8%) transfected cells displayed significantly lower numbers of E4-positive cells than E8- (2.3%), E8 SD mt (3.6%), or E8 RPR mt (2.0%) transfected cells. This indicates that the loss of functional E8^E2 induces expression of the late E4 protein. HPV E4 expression in undifferentiated human cells has been shown to induce an arrest in the G2 phase of the cell cycle (27 - 29). We, therefore, quantified the DNA content in E4-negative (E4−) and E4-positive (E4+) cells by flow cytometry. The most obvious changes were the appearance of a small sub-G0/G1 peak in E4+ cells, indicating cells with fragmented DNA indicative for cell death and a shift in the distribution of the different cell cycle phases (Fig. S2). A quantification of the cells in the different cell cycle phases (G0/G1; S and G2/M) revealed no consistent changes between E4-negative and E4-positive cells in the G0/G1 phase (Fig. 8A). In contrast, the fraction of E4-positive cells in S-phase was significantly reduced and in the G2/M phase significantly increased (Fig. 8B and C). These data are consistent with the idea that the expression of mE4 induces a G2 arrest similar to HPV E4 proteins. However, it is also possible that this reflects a greatly prolonged G2 phase. The quantification of cells in sub G0/G1 5 d p.t. indicated that mE4-expressing cells show an increase in this cell population compared to mE4-negative cells. The reduced DNA content points to the possibility that these cells are undergoing cell death. These differences were statistically significant for WT, E8-, E8 RPR mt, E8 SD mt and showed a trend for mE4 and E8 Stop mt-transfected cells (Fig. 8D). Interestingly, the shift in cell cycle occurred also when the mE4 expression vector was used, suggesting that mE4 expression is a major contributor to these effects. High-level E4 expression from wt HPV genomes in tissue culture requires keratinocyte differentiation (32, 33). To understand if the loss E8^E2 overcomes the need for cellular differentiation, we stained E4-positive cells after transfection of WT, E8-, E8 RPR mt, or E8 SD mt genomes with keratin 14, a marker for basal-like keratinocytes, and keratin 10, a marker for suprabasal keratinocytes (34). Consistent with the culture of NMTK in serum-free medium and a low concentration of Ca^2+^ to preserve their basal-like phenotype, almost all cells were positive for keratin 14 and only very few cells expressed keratin 10 (Fig. 9A and B). All E4-positive cells in WT and E8^E2 mt transfections were positive for keratin 14 (Fig. 9A), whereas only 14.5% (WT), 6.1 (E8-), 12.5% (E8 RPR mt), and 12.5% (E8 SD mt) of the E4-positive cells were positive for keratin 10 (Fig. 9B). This suggests that E4 expression in cultured mouse keratinocytes occurs mainly in cells with a basal-like phenotype. To further evaluate if the loss of E8^E2 induces the late phase in cultured NMTK, we analyzed transcripts for the early *E6* and *E7* genes derived from the early P7503 and P360 promoters and the *E1^E4* and *URR^E4* transcripts that are mainly transcribed from the late P7107 and P533 promoters (35). To avoid the detection of genomes in the case of *E6* and *E7* (as no splicing occurs in these genes), mRNA was isolated via polyA+ selection and then subjected to qPCR (Fig. 10). *E6* levels were 2.5-fold increased 3 d p.t. and sixfold 6 d p.t. in E8- compared to WT-transfected cells. Similar increases were observed for *E7* levels. However, *E1^E4* levels were increased 16.4-fold (3 d p.t.) and 17.6-fold (6 d p.t.) and *URR^E4* levels were activated 29.6-fold (3 d p.t.) and 33.8-fold (6 d p.t.). This strongly indicates that the loss of mE8^E2 activates viral late transcription to a much greater extent than viral early transcription consistent with a switch to the productive phase.

## DISCUSSION

Papillomaviruses replicate and propagate in keratinocytes in a highly spatially and temporally controlled manner tightly associated with stratified squamous epithelial differentiation. Productive genome replication, capsid protein expression, and synthesis of infectious progeny only occur in the suprabasal layers of the epithelium in keratinocytes that have entered their terminal differentiation program. Despite intensive research, the events required for HPV propagation are only partially understood. A hallmark for the start of productive replication in suprabasal keratinocytes is the activation of the viral late promoter in *E7* and genome amplification leading to the abundant expression of the viral E4 protein (32, 33, 36, 37). Current models suggest that the HR-HPV late promoter is activated by differentiation-regulated transcription factors such as C/EBPβ which, in turn, induces genome amplification (38 - 42).

Previous studies have shown that the knockout of E8^E2 in different HPV types results in increased genome replication and viral gene expression in undifferentiated cells (13 - 19). Consistent with this, mutation of the putative start codon for *E8^E2* in the MmuPV1 genome (E8-) also resulted in increased viral transcription in undifferentiated NMTK (26). Surprisingly, despite being more transcriptionally active, MmuPV1 E8- genomes failed to induce warts or be maintained in the epithelium of T-cell deficient mice (26). To validate that this phenotype was caused by a lack of E8^E2 expression, we generated additional mutations predicted disrupt E8^E2 expression. Mutation of the conserved *E8* splice donor site (E8 SD mt) greatly increased viral gene expression in undifferentiated NMTK consistent with the spliced *E8^E2* transcript being responsible for the expression of a repressor protein. Surprisingly, the introduction of a translation termination codon at codon 9 of *E8* did not increase viral gene expression. Transcript analyses revealed that this mutation additionally created a novel splice donor at nt. 1116 leading to a transcript in which E8 residues 9–11, and thus the stop codon, are removed. The functional analysis mE8^E2 d9-11 indicated that this protein acts as a transcriptional repressor comparable to wt mE8^E2. Thus, mE8^E2 d9-11 most likely compensates for mE8^E2 expression and prevents the induction of viral gene expression. Consistent with E8 residues 9–11 not being important for repression activity, G9 and A10 are not conserved and conserved S11 is followed by three consecutive serines which may compensate for the loss of S11 (Fig. 2A). Mutation of residues 8–12 in HPV31 E8^E2 also had no impact on transcriptional repression supporting the idea that this part of E8 is not important for the repression activity (43).

HR-HPV16 and 31 and cutaneous HPV8 E8^E2 proteins interact via conserved residues in E8 (K5/W6/K7 in HPV16 and 31; K2/L3/K4 in HPV8) with NCoR/SMRT complexes to inhibit transcription and replication (9). Consistent with this, mutation of K2/L3/K4 residues in mE8^E2 attenuates repression of transcription and replication reporters in human and mouse cells and also increases transcription from MmuPV1 genomes in keratinocytes. Furthermore, mE8^E2 interacts with GPS2, HDAC3, TBLR1, and TBL1 which are different components of NCoR/SMRT complexes in co-IP assays in a KLK-dependent manner, and siRNA knockdown experiments confirm that NCoR and SMRT or HDAC3 and TBLR1 relieve repression in an E8^E2-dependent manner. Notably, the knockdown of GPS2 on its own had no impact on mE8^E2´s repression activity. This suggests that repression is largely independent from GPS2 and also that GPS2 cannot be the unknown direct interactor of E8^E2 proteins in NCoR/SMRT complexes. These data indicate that the use of NCoR/SMRT complexes by E8^E2 proteins to limit viral gene expression and replication is highly conserved among PV and also necessary for tumor formation *in vivo*. This interaction, which is broadly conserved, potentially could be targeted by small molecules as a therapeutic for infection by diverse HPV types.

E8 SD mt and E8 RPR mt genomes displayed increased viral gene expression in cultured NMTK but failed to induce tail warts or maintain viral gene expression and genomes at infected sites confirming the phenotype of the MmuPV1 E8- genome (26). This strongly suggests that loss of E8^E2 activity by a complete knockout (E8-, E8 SD mt) or by abolishing its repression activity (E8 RPR mt) is responsible for the increased gene expression from viral genomes and the failure to induce warts *in vivo*.

Remarkably, MmuPV1 E8^E2 mt genomes displayed an increased number of cells expressing the late viral E4 protein in monolayer culture. Expression of HPV E4 proteins has been shown to induce a G2 arrest in monolayer cultures independent from other viral gene products (27 - 29). Our analyses reveal that the expression of mE4 from a vector or from wt or mt MmuPV1 genomes in normal murine keratinocytes induces a shift in the cell cycle consistent with a G2 arrest. Remarkably, almost all E4-positive cells stained positive for keratin 14, a marker for basal keratinocytes, but were only very rarely positive for keratin 10, a marker for suprabasal keratinocytes. This strongly indicates that the loss of mE8^E2 greatly increases the probability to induce vegetative viral genome replication independent from keratinocyte differentiation. This is further supported by the finding that the late *E1^E4* and *URR^E4* transcripts are induced to a much greater extent than the early *E6* and *E7* transcripts from E8- genomes. The induction of E4 protein expression from HPV31 E8^E2 mt genomes in undifferentiated keratinocytes may also explain the inability to stably maintain these genomes as autonomously replicating elements. However, replicating HPV16 E8^E2 mt genomes can be maintained in keratinocytes, and the number of E4-positive cells is increased only in the suprabasal layers in organotypic cultures (18). This suggests that HPV16, in contrast to MmuPV1, may require, in addition to the loss of E8^E2, a second differentiation-dependent signal to initiate productive replication. Similarly, HPV49 E8- genomes can be stably maintained at high extrachromosomal copy numbers in undifferentiated keratinocytes suggesting that HPV49 may also require a differentiation signal to efficiently express E4 or that the HPV49 E4 protein has different activities than the mE4 protein. However, since *E6* and *E7* levels are also induced upon a loss of E8^E2 (Fig. 10) (14 - 18 - 20 - 20), it is also possible that not only E4 but also the activities of E6 and E7 determine the fate of E8^E2 mt genomes.

Cell cycle analyses indicated that mE4-expressing keratinocytes are preferentially in the G2/M compared to the S and G1/G0 phase. This is consistent with findings that HPV E4 proteins induce a G2 arrest in undifferentiated cells (27 - 29). HR-HPV genome amplification and E4 protein expression in differentiated keratinocytes preferentially take place in the G2 phase (44, 45). These cells then exit the cell cycle and do not undergo cell division before being shed from the epithelium (44, 45). Based on the tissue culture data, we propose that the infection of basal layer keratinocytes with MmuPV1 E8^E2 mt genomes greatly enhances the probability of an immediate switch to the productive replication phase leading to genome amplification which induces high levels of E4 protein. The resulting shift to the G2 phase prevents the cells from dividing. This would prevent an efficient expansion of infected cells in the basal layer and, in turn, the development of warts. Furthermore, the DNA content analysis provided evidence that a small fraction of E4-positive cells undergoes cell death which is consistent with the induction of apoptosis by HPV16 E4 upon its expression in HeLa cells (46). This would further diminish the number of infected cells over time and could account for the complete absence of viral RNA and DNA at E8- genome challenge sites (26).

We have recently hypothesized that HPV E8^E2 is part of a copy number control mechanism required to maintain viral genomes in dividing cells at a low but relatively constant copy number (47). The results presented here suggest that the primary function of MmuPV1 E8^E2 is to prevent the initiation of productive replication in basal-like cells in order to allow for lateral expansion of infected cells and thus maximizing virus production. As pointed out above, HPV16 E8^E2 mt genomes express high levels of E4 only in suprabasal, differentiated cells indicating that HPV16 uses additional mechanisms to prevent productive replication in undifferentiated cells (18).

We have previously speculated that E8^E2, due to its high conservation, might represent an attractive anti-viral target even in settings without functional T-cells (26). In the light of the data presented here, an interference with E8^E2 activity in wt-induced lesions might have the unintended consequence of enhancing productive replication and virion production. However, it is also possible that an inappropriate induction of the viral late phase in cells not destined for this might interfere with cell survival or lesion growth and, thus, have a therapeutic effect. While the tissue culture data for HPV16 argue against a clinical benefit of an interference with E8^E2, our mouse studies suggest that the outcome might be therapeutic *in vivo*. However, one should also consider that an interference with E8^E2 activity might induce malignant tumors from HPV49-infected cells (16). Nevertheless, different patient groups have been identified which display generalized wart or papilloma growth, and recent data suggest that this is often due to inherited immunodeficiencies (48). Since E8^E2 is highly conserved and required for wart growth in T-cell deficient mice, such patients might benefit from an inhibition of E8^E2. Taken together, it might be worthwhile to identify means to interfere with E8’s interaction with the co-repressor complex and evaluate its potential for the treatment of mucosal and cutaneous HPV infections.

The high conservation of E8^E2 and the observation that the inactivation of E8^E2 facilitates the switch to the productive phase make it likely that the inhibition by E8^E2 needs to be at least partially overcome to allow the differentiation-dependent switch in wt-infected cells. Data obtained for HPV16 and 31 do not indicate that *E8^E2* expression is transcriptionally downregulated upon differentiation (19, 47). However, it has been proposed that the activation of the late promoter leads to increased E2 protein levels which might outcompete E8^E2 protein levels (49, 50). Future studies are required to understand how E2 and E8^E2 regulate productive genome amplification and late gene expression in differentiating cells.

## MATERIALS AND METHODS

### Recombinant plasmids

Plasmids pSG HPV16 E8^E2-HA, pSG mE8^E2, pSG mE8^E2-HA, pSG mE2, pSG mE1, pC18-Sp1-luc, pGL mURR/E1-luc, pUC57 mE1^E4^L1, pUC57 mURR^E4, pAsylum-MmuPV1, and pAsylum-MmuPV1 E8- have been previously described (49, 50). Plasmids pAsylum-MmuPV1-E8 RPR mt, E8 Stop, and E8 SD mt were constructed by overlap extension PCR and replacement of restriction fragments. In plasmids pSG mE8^E2 RPR mt and pSG mE8^E2-HA RPR mt, the codons for E8 K2/L3/K4 residues were mutated to AGA CCC AGG resulting in E8 *R2*/P3/R4 by overlap extension PCR. Plasmids pSG mE8^E2 d9-11 and pSG mE1 G126V were generated by overlap extension PCR. To construct plasmid pSG 3xHA-mE1^E4, the mE1^E4 coding sequence was amplified by PCR and cloned into pSG-3xHA. To generate psYFP-HDAC3, the coding sequence for human HDAC3 was PCR-amplified from pcDNA3.1 hsHDAC3-FLAG (51, 52) and inserted into pSYFP2-C1, a gift from Dorus Gadella [Addgene plasmid # 22878 (53)]. To generate pGPS2-sYFP and psYFP-TBLR1, the coding sequences of human GPS2 or TBLR1, respectively, were amplified from human cDNA and inserted into pSYFP2-C1. Plasmid pCI-neo-Rluc is based on pCI-neo (Promega) and expresses a codon-optimized version of renilla luciferase that was amplified from plasmid pGL4.72 (Promega). All inserts were verified by DNA sequencing, and MmuPV1 mutant genomes were completely sequenced to ensure that no additional changes were introduced during the cloning procedure.

### Cell culture

C33A and HeLa cells were maintained in Dulbecco’s modified Eagle’s medium supplemented with 10% fetal bovine serum and antibiotics. Isolation and transfection of NMTKs were essentially done as described previously (26). NMTKs were grown in keratinocyte serum-free medium supplemented with 50 µM Ca^2+^, epidermal growth factor, and bovine pituitary extract (Gibco) as described previously (26).

### Luciferase reporter assays

C33A cells (7 × 10^4^) or primary mouse keratinocytes were transfected using Fugene HD (Promega) and DNA amounts indicated in the figure legends. Firefly and renilla luciferase activities were determined 48 h post transfection using a TriStar2 S LB 942 multimode plate reader (Berthold Technologies).

### SiRNA

SiRNAs targeting human NCOR1, NCOR2 (SMRT), HDAC3, or TBLR1 have been previously described (9). To target, human GPS2 ON-TARGETplus SMARTpool hGPS2 (Dharmacon, Thermo Scientific; M-004329-01-0005), mouse Ncor1 ON-TARGETplus SMARTpool mNcor1 (Dharmacon, Thermo Scientific; L-058556-00-0005), or mouse Ncor2 ON-TARGETplus SMARTpool mNcor2 (Dharmacon, Thermo Scientific; L-045364-00-0005) was used.

### RNA analysis

Total RNA was isolated from cultured mouse keratinocytes or human cells using the RNeasy minikit (Qiagen). MRNA was enriched from total RNA using the RNeasy Pure mRNA Bead Kit (Qiagen). RNA (1 µg) was reverse transcribed using the QuantiTect reverse transcription kit (Qiagen), and 25 ng of cDNA was analyzed in duplicates by qPCR in a LightCycler 480 (Roche Applied Science) for viral and cellular transcripts using LightCycler 480 SYBR green I master mix (Roche Applied Science) and 0.3 mM of primer pairs for MmuPV1 *URR^E4*, MmuPV1 *E1^E4*, or MmuPV1 *E8^E2* (MMPV1 1055 F gaaagagcaggagacggttg, mMPV1 3169 R TTTTTGATGCCCTTCTTTGG), MmuPV1 *E6* (mE6 7 F ATCGGCAAAGGCTACACTCTC, mE6 194 R CTGCGGCACACAATACAAGC), MmuPV1 *E7* (mE7 491 F GTGAGCCTGACCTACCCGAT, mE7 622 R GTCGCAGCAAAAGCAGGTTG), or *PGK1* which was used as a cellular reference gene (26). To determine the effect of siRNA knockdowns, primers (in 5′−3′ direction) to detect human NCOR1 (hsNCOR 1715 F: GAAAGACTGCCAACAGTCAGG, hsNCOR 1883 R: CATCGAGAGGTCTCCACAGG), human NCOR2 (hsSMRT 1428 F: GCACGAGGTGTCAGAGATCA, hsSMRT 1653 R: GAACTTCTCCCGGAAGGTCT), human TBLR1 (hsTBLR1 E11 F: CCAGCATTGGATGTTGATTG, hsTBLR1 E13 R: ATGTGCTTGCAAATCATGGA), human HDAC3 (hsHDAC3 E1 F: ACGTGGGCAACTTCCACTAC, hsHDAC3 E3 R: GACTCTTGGTGAAGCCTTGC), human GPS2 (hsGPS2 F: GAGAAGCACCAGCTTTTCCTGC, hsGPS2 R: GAACAGTCAGGCTCTGCTGGTA), mouse Ncor1 (mNcor1 F: CTGGTCTTTCAGCCACCATT, mNcor1 R: CCTTCATTGGATCCTCCATC), or mouse Ncor2 (mSmrt F: GGGTAAATATGACCAGTGGGAAGAG, mSmrt R: TGGCATTCAGAGGGTTAAAAGC) were used. LightCycler 480 software version 1.5 was used for quantification, the second derivate/max analysis was chosen to obtain Cq values, and melting curves were analyzed to ensure the measurement of a single amplicon. Standard curves for *E1^E4*, *URR^E4*, *E8^E2,* and *PGK1* were generated using serial dilutions of plasmids containing cloned cDNAs. *E8^E2* cDNAs for sequencing were amplified with MmuPV1 1055 F and MmuPV1 3312 R (atgcaggtttgtcgttctcc), gel-purified and sequenced.

### Immunoblot and co-immunoprecipitation analysis

C33A or HeLa cells (2.5 × 10^6^) were seeded in 100 mm cell culture dishes. The next day, they were transfected; 48 h later, they were harvested; and then lysed in IP-buffer (50 mM HEPES pH 7.9, 150 mM NaCl, 0.3% (v/v) Igepal 630, 1 mM DTT, protease, and phosphatase inhibitors). Immunoprecipitation was carried out using magnetic anti-HA-beads (Miltenyi Biotech). Beads were washed with IP-buffer using μMACS columns and μMACS Separator (Miltenyi Biotech). Bound proteins were eluted in elution buffer [50 mM Tris HCl (pH 6.8), 50 mM DTT, 1% SDS, 1 mM EDTA, 0.005% bromephenol blue, 10% glycerol] heated to 95°C and analyzed by immunoblotting.

Separated proteins were transferred onto a 0.22 mm nitrocellulose membrane (Protran) in 10 mM CAPS and 10% (v/v) methanol (pH 10.3). Membranes were blocked in 5% milk in PBS with 0.1% Tween20. Membranes were incubated overnight with the primary Abs at 4°C. The following primary antibodies were used at the indicated dilution: HA-Tag (Cell signaling, rabbit mAb, C29F4, #3724,1:1,000), sYFP (Clontech, mouse mAb, JL-8, from # 632381, 1:1,000), HDAC3 (abcam, rabbit mAb, ab76295, 1:1,000), and TBL1 (Santa Cruz BT, mouse mAb, H-3, sc-137006, 1:750). Secondary fluorescence-labeled Abs (1:15,000; LI-COR) were added for 1 h, and signals were detected using the Odyssey Fc Infrared Imaging System (LI-COR Biosciences).

### Immunofluorescence analysis

For immunofluorescence analyses, cells were seeded on 35 mm glass bottom dishes (MatTek) and transfected the next day with expression plasmids or MmuPV1 genomes. Cells were fixed 48 h later by the addition of 4% paraformaldehyde and incubated for 15 min at room temperature. Afterward, cells were washed three times with PBS and incubated at RT for 1 h in blocking buffer (1 × PBS, 5% (v/v) normal goat and donkey serum, 0.3% (v/v) Triton-X 100) followed by incubation in primary antibody diluted in antibody-dilution-buffer (1 × PBS, 1% (w/v) BSA Fraction V, 0.3% (v/v) Triton-X 100) at 4°C overnight. The next day, dishes were washed three times in PBS and then incubated with the secondary fluorescence-labeled antibody (anti-mouse-488/555, anti-rabbit-488/555, anti-chicken-488) for 1 h at room temperature. After three PBS washing steps, DAPI solution was added for 30 s, followed by three additional washing steps with PBS. Samples were analyzed with a Zeiss Axio Observer microscope and the appropriate filter sets, and 63× magnification was used in combination with a Zeiss Apotome. The rabbit polyclonal anti-MmuPV1 E4 serum was used at a 1:3000 dilution and has been previously described (22). Anti-Keratin 10 (mouse monoclonal antibody, DEK10, DAKO, dilution 1:200, #154736) and anti-Keratin 14 (chicken polyclonal antibody, BioLegend, dilution 1:300, #906004) were commercially obtained.

### Flow cytometry

NMTKs were seeded in 6-well dishes and transfected with expression plasmids or different MmuPV1 genomes. Cells were harvested by trypsinization (5 min, 600 × g, 20°C). The pellet was resuspended in FACS buffer (1 × PBS, 1% FCS) and then centrifuged. Pelleted cells were fixed with 4% PFA for 10 min at 37°C shaking and centrifuged for 10 min at 600 g. The pellet was resuspended in FACS buffer and centrifuged. To permeabilize the cells, the pellet was resuspended in 100 µL of permeabilization buffer [FACS Buffer + 0.1% (v/v) Triton-X-100] at RT for 5 min, and the cells were washed twice with FACS buffer. After centrifuging, the pellet was resuspended in 150-µL blocking buffer (FACS buffer + 10% FCS) and incubated at RT for 30 min. Cells were collected by centrifugation, and the primary antibody was added in FACS buffer and incubated for 1 h at 4°C (rabbit-anti-E4, 1:400). After that, the cells were washed twice in FACS buffer, stained with the secondary antibody [Goat anti-rabbit IgG (H + L) Cross-Adsorbed Secondary Antibody, Alexa Fluor 405, 1:1000, Thermo Fisher Scientific] for 1 h at 4°C, and washed again before staining with propidium iodide (50 µg/mL) supplemented with 0.33 mg/mL RNAse A and incubated for 30 min at 37°C. Cells were then analyzed with MACS Quant.

### Animal experiments

All animal studies were carried out in accordance with the recommendations in the Guide for the Care and Use of Laboratory Animals of the National Institutes of Health and with the prior approval of the Animal Care and Use Committee of Johns Hopkins University. Athymic nude female mice (ENVIGO, strain 069) 2–3 months of age were challenged with either WT or mutant MmuPV1 DNA. For MmuPV1 DNA, plasmids containing the WT, E8 RPR mt, E8 SD mt, or E8 Stop mt MmuPV1 genomes inserted at XbaI in the pAsylum1 vector (pMmuPV1 kindly provided by C. Buck, NCI) were prepared using Qiagen HiSpeed plasmid maxikit (catalog no. 12663; Qiagen). For tail injection, MmuPV1 DNA was linearized by digestion with XbaI (NEB). The tails of anesthetized mice were subjected to epithelial abrasion by rubbing with autoclaved sand paper (3M Sandblaster Pro 150 medium). Three days after the initial injury, a total of 30 µg of linearized plasmid was injected into five sites underneath scabs that developed from the skin trauma (26).

### qPCR detection of MmuPV1 DNA and transcripts in mouse tissue

All mice were sacrificed 4–5 months after challenge. Tail tissues were excised into 0.3 mL of TRIzol (#15596026, Thermo Fisher) and lysed using gentleMACS dissociator. MmuPV1 transcripts were detected by qPCR as described previously (26). For detection and identification of WT and mutated MmuPV1DNA, tail tissues were homogenized in 1 mL of DPBS with 0.8 NaCl using gentleMACS dissociator. DNA was extracted from the lysate using Quick-DNA Universal Kit (#D4068, Zymo Research). E8 sequences were amplified by PCR with forward primer 5′-GTGGATCAGGGAAATTCCTTGGC-3′ and reverse primer 5′-AGGTTTAAGGAGGCATTTGGGTG-3′. The PCRs were purified using QIAquick PCR Purification Kit (#28104, Qiagen). The presence of either WT or mutated MmuPV1 sequences was verified by Sanger sequencing of amplicons using primer GTATGCCGCGACACCTAAGA.

## SUPPLEMENTAL MATERIAL

Figure S1 mbio.00696-23-s0001.tif Validation of the specificity of the anti-ME4 antibody.

Figure S2 mbio.00696-23-s0002.tif Flow cytometry plots demonstrating cell cycle profiles in mE4-positive and -negative cells.

ASM does not own the copyrights to Supplemental Material that may be linked to, or accessed through, an article. The authors have granted ASM a non-exclusive, world-wide license to publish the Supplemental Material files. Please contact the corresponding author directly for reuse.
