# Supplementary figures and images for: Mus musculus papillomavirus 1 E8^E2 represses expression of late protein E4 in basal-like keratinocytes via NCoR/SMRT-HDAC3 co-repressor complexes to enable wart formation in vivo
Source: mBio. 2023 Jun 29;14(4):e00696-23. doi: 10.1128/mbio.00696-23 (PMC10470772; doi:10.1128/mbio.00696-23)

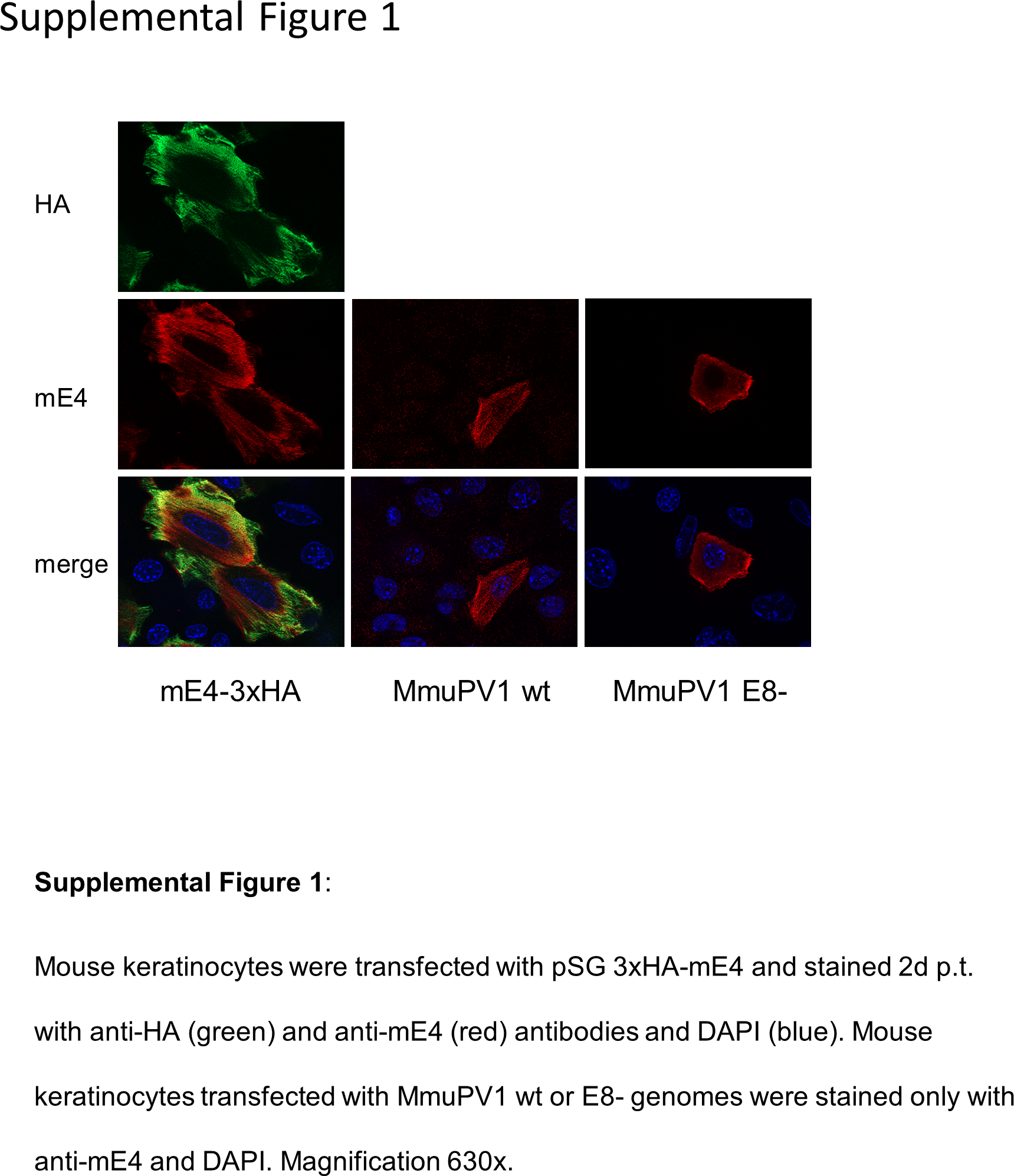

Supplement: Figure S1 — Validation of the specificity of the anti-ME4 antibody. [file mbio.00696-23-s0001.tif]

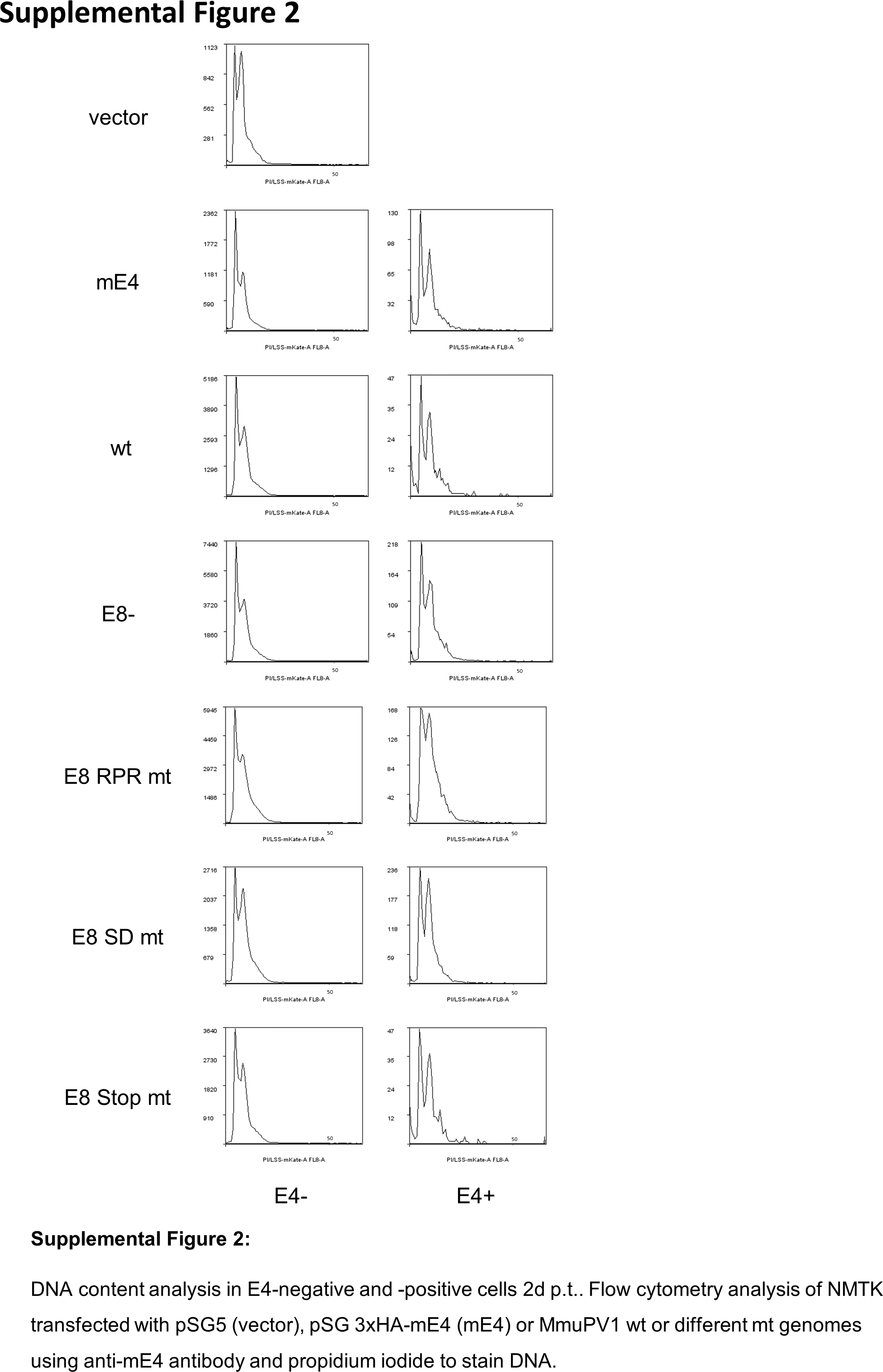

Supplement: Figure S2 — Flow cytometry plots demonstrating cell cycle profiles in mE4-positive and -negative cells. [file mbio.00696-23-s0002.tif]
